# Single‐Crystal‐to‐Single‐Crystal Transformation in a Thermally Stable All‐Inorganic Polyoxoniobate Framework Boosts Lithium Ion Battery Anode Performance

**DOI:** 10.1002/anie.202506533

**Published:** 2025-06-03

**Authors:** Cai Sun, Jian‐Ping Chen, Yan‐Lan Wu, Yi‐Ying Li, Xin‐Xiong Li, Ping‐Wei Cai, Carsten Streb, Shou‐Tian Zheng

**Affiliations:** ^1^ Fujian Provincial Key Laboratory of Advanced Inorganic Oxygenated‐Materials College of Chemistry Fuzhou University Fuzhou Fujian 350108 China; ^2^ Department of Chemistry Johannes Gutenberg University Mainz Duesbergweg 10–14 55128 Mainz Germany

**Keywords:** Capacity enhancement, Li‐Ion battery, Polyoxometalate, Polyoxoniobate, SCSC Transformation

## Abstract

Niobium oxides are considered as promising anode materials for lithium‐ion batteries (LIBs) due to their excellent rate‐performance. However, the practical application is hindered by their limited specific capacity. In this work, we report the first example of an all‐inorganic two‐dimensional (2D) niobate framework as anode material for LIBs. The title compound is based on antimony‐linked bivanadyl‐capped *α*‐Keggin polyoxoniobates as secondary building units. The compound undergoes a unique single‐crystal‐to‐single‐crystal (SCSC) transformation triggered by formic acid which results in the migration of a {VO} unit into the framework interlayer. This results in a 34% increase of the specific capacity, reaching 519 mAh g^−1^ at 0.1 A g^−1^, thereby surpassing most Nb‐based LIB anode materials. Experimental and theoretical calculations reveal that the SCSC transformation exposes more Li‐binding sites in the framework, and reduces the interlayer Li‐ion diffusion barrier, leading to a capacity increase. This work presents the first example of a SCSC transformation leading to enhanced LIB performance and offers atomic‐level insights into the design of advanced LIB anode materials.

## Graphical abstract

A single‐crystal‐to‐single‐crystal transformation in the first antimony‐bridged all‐inorganic two‐dimensional (2D) polyoxoniobate framework exposes additional Li‐binding sites and reduces the interlayer Li‐ion diffusion barrier, resulting in a significant increase in capacity of Li‐ion batteries.

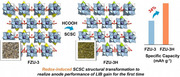

## Introduction

The continuously growing global energy demand has significantly accelerated research into high‐performance, cost‐effective, and sustainable energy storage technologies.^[^ ^1^, ^2^, ^3^, ^4^ ^]^ Among the various options, lithium‐ion batteries (LIBs) currently dominate the energy storage market due to their high operating potential and high energy/power density.^[^ ^5^, ^6^, ^7^ ^]^ To date, carbon‐based materials have been regarded as the standard commercial anode of LIBs, primarily owing to their low cost and high safety.^[^ ^8^, ^9^ ^]^ However, their relatively low specific capacity (theoretical value of 372 mAh g^−1^) is still not satisfactory for the application of electric vehicles. Accordingly, it is of great significance to develop high‐capacity anode materials to achieve new performance benchmarks.

Over the past decades, various materials classes including metals, metal oxides, and metal sulfides have been explored as promising anode materials to enhance LIBs performance due to their conversion/alloying reaction mechanism.^[^ ^10^, ^11^, ^12^ ^]^ Orthorhombic Nb_2_O_5_, known for its rapid energy storage capabilities, has emerged as a high‐rate anode material for LIBs, demonstrating significant potential for future applications.^[^ ^13^, ^14^ ^]^ However, despite the implementation of various strategies, such as doping, surface functionalization, and defect engineering,^[^ ^15^, ^16^, ^17^ ^]^ the capacity of Nb_2_O_5_ remains limited to about 200 mAh g^−1^, which falls short of practical application requirements. The primary reason is the lack of sufficient understanding of the relationship between the structure and capacity of niobium‐based materials. Thus, developing new strategies to obtain high‐rate Nb‐based materials with increased capacity is crucial for improving battery performance.^[^ ^18^, ^19^, ^20^ ^]^

Polyoxoniobates (PONbs), a class of polyoxoanions composed of Nb and O atoms with well‐defined structures,^[^ ^21^, ^22^ ^]^ are promising anode materials for LIBs due to their ability to accommodate large numbers of electrons and ions while maintaining structural stability. Moreover, their structural tunability and modifiability allow for single‐crystal‐to‐single‐crystal (SCSC) transformations driven by external stimuli,^[^ ^23^, ^24^ ^]^ offering a rare opportunity to investigate the relationship between structure and capacity with atomic‐level precision. Especially, the use of SCSC transformations to control the performance of LIB performance has not yet been reported. However, there is only a limited number of PONb structure types accessible, owing to synthetic challenges caused by narrow and strongly basic operating pH regions. In addition, low solubility and low reactivity of many niobate species add to this challenge. Further, there are only very few examples of all‐inorganic extended PONb frameworks bridged through metal linkers which could be viable prototypes for LIB studies.^[^ ^25^ ^]^

Here, we report a novel and rare all‐inorganic two‐dimensional (2D) PONb framework, Li_2_K_5_[Sb(H_2_O)][GeNb_12_O_40_(V^V^O)_2_]·8H_2_O (FZU‐3), which is constructed from SBUs of bivanadyl‐capped *α*‑Keggin PONb, [GeNb_12_O_40_(V^V^O)_2_] (denoted as GeNb_12_V_2_), bridged by [Sb(H_2_O)]^3+^ antimony complex linker. The framework undergoes a rapid SCSC structural transformation, triggered by HCOOH within just 5 min to form a new 2D PONb framework, H_1.5_LiK_2_V^V^_1.5_[Sb(H_2_O)][GeNb_12_O_40_(V^IV^O)_0.5_]·8H_2_O (FZU‐3H), involving the dissociation and migration of one and half capped {V^V^O} group into the interlayer of the PONb framework, as well as the reduction of the remaining {V^V^O} cap to {V^IV^O}. When using these frameworks as anodes for LIBs, FZU‐3H exhibits higher specific capacity and rate capability compared to FZU‐3, even outperforming most of the reported niobium‐based anode materials of LIBs. To our knowledge, this is the first successful application of SCSC structural transformation to realize enhanced LIB anode performance. The high‐quality single crystal X‐ray diffraction (SCXRD) data offer atomic‐level insights into the behavior of enhanced capacity. The migration of the capped V^(V)^ ion leads to structural change with an increased void space within the framework, enhancing the ability to accommodate more Li^+^ ions, contributing to a capacitive storage mechanism, particularly in the low‐voltage region. Computational simulations further reveal that the structural transformation effectively reduces the activation energy of Li^+^ ion diffusion, accelerates Li^+^ ion diffusion, and enhances the capacity.

## Results and Discussion

The pale‐brown block‐shaped crystal of FZU‐3 was obtained by a hydrothermal reaction of K_7_HNb_6_O_9_·13H_2_O, Sb_2_O_3_, V_2_O_5_, GeO_2_, and LiCl in H_2_O at 160 °C for 72 h. The phase purity of FZU‐3 was confirmed by powder XRD (PXRD, Figure S1) and inductively coupled plasma optical emission spectrometry (ICP‐OES) analysis (see Experimental Section in the Supporting Information). Thermogravimetric analysis (Figure S2), and in situ variable‐temperature PXRD data showed that the framework was thermally stable at least up to 500 °C (Figure S3). Single‐crystal X‐ray diffraction (SXRD) analysis shows that FZU‐3 crystallizes in the tetragonal space group *I*4/mmm, exhibiting an inorganic Sb‐bridged 2D PONb framework based on bivanadyl‐capped Keggin polyoxoanion GeNb_12_V_2_ as secondary building units (SBUs). It is noteworthy that, although both infinitely extended metal‐bridged PONb frameworks and Sb‐containing PONbs have been known,^[^ ^26^, ^27^, ^28^, ^29^, ^30^, ^31^, ^32^ ^]^ FZU‐3 represents the first example of an infinitely extended Sb‐bridged PONb framework.^[^ ^33^ ^]^

**Figure 1:**
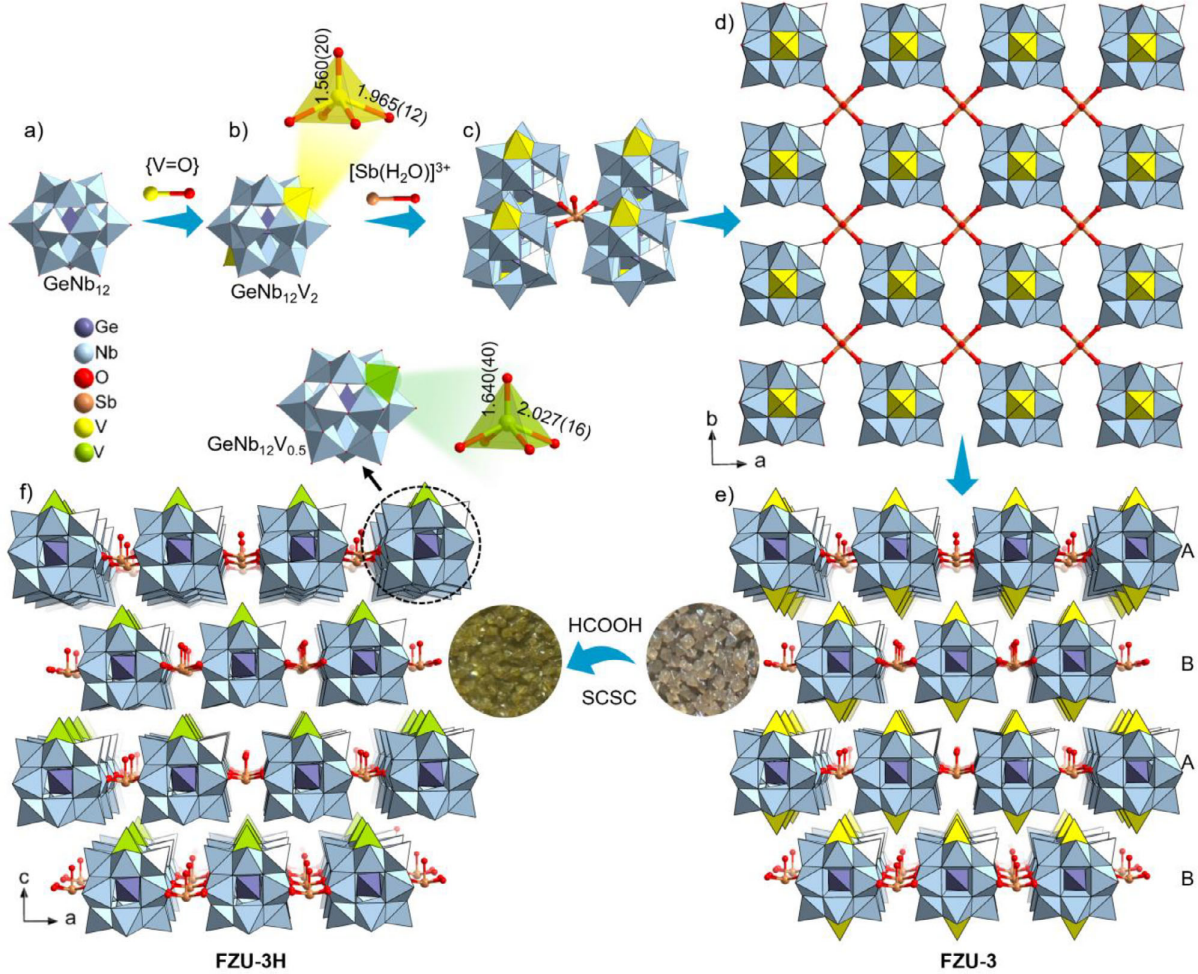
All‐inorganic 2D frameworks of FZU‐3. a) GeNb_12_ unit. b) GeNb_12_V_2_ SBU. c) Sb‐bridged GeNb_12_V_2_ tetramer. d) 2D layer of FZU‐3. e, f) View of the SCSC transformation between FZU‐3 and FZU‐3H.

As shown in Figure 1a,b, the GeNb_12_V_2_ SBU can be derived from known bivanadyl‐capped Keggin polyoxoanion {PV_2_Nb_12_O_42_} by the substitution of its central P atom with a Ge atom.^[^ ^26^ ^]^ The capping VO_5_ has a short axial V═O bond of 1.560(20) Å and four equatorial V─O bonds of 1.965(12) Å. Bond‐valence‐sum (BVS) calculations indicate that these capping vanadium atoms are in the V^V^ oxidation state (Table S2). Interestingly, due to the positional disorder of the GeO_4_ tetrahedron, the GeNb_12_V^V^_2_ SBU possesses *D*_4h_ symmetry, with the *C*_4_ axis passing through the two capping {V═O} units (Figure S4). At this point, four Nb2‐centered octahedra are located on the equatorial plane, while eight Nb1‐centered octahedra are positioned at the top and bottom.

Additionally, the [Sb(H_2_O)]^3+^ units, acting as linkers, connect four adjacent GeNb_12_V_2_ SBUs and further form a tetragonal reticular 2D framework layer along *ab* plane (Figure 1c). Inside a tetrameric fragment, a Sb^3+^ ion coordinates with one H_2_O with Sb─O bond length of 1.960(40) Å, and four terminal oxo O_t_ atoms from the Nb2‐centered octahedra with Sb─O bond length of 2.218(17) Å, forming a 5‐coordinate umbrella‐like square pyramidal SbO_5_ with O_t_─Sb─O bond angle of 81.44(12)° (Figure S5). This umbrella‐like coordination configuration arises from the lone pair electrons of Sb^3+^ ions. Finally, the two adjacent 2D framework layers (Figure 1d) extend infinitely in the *c*‐direction through an ABAB stacking pattern (Figure 1e).

**Figure 2:**
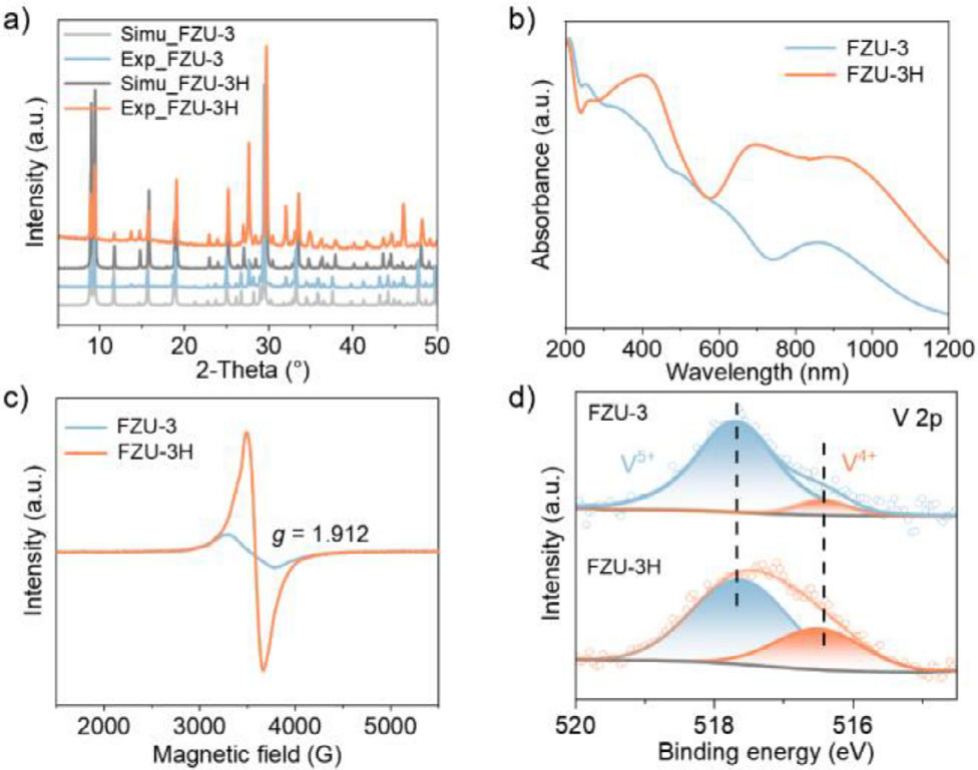
a) PXRD patterns, b) UV–vis spectra, c) EPR spectra, and d) V 2p XPS core level spectra of FZU‐3 and FZU‐3H.

Immersion of crystalline samples of FZU‐3 in HCOOH solution for 5 min at ambient temperature results in a SCSC transformation: the pale‐brown crystalline FZU‐3 changes to yellowish‐green single crystals of FZU‐3H (Figure 1f). High‐quality SXRD and ICP‐OES data of FZU‐3H provide atomic‐level insights into the HCOOH‐induced reactivity. FZU‐3H maintains a similar all‐inorganic 2D framework to FZU‐3. Specifically, the bivanadyl‐capped Keggin‐type GeNb_12_V_2_ SBU transforms into a monovanadyl‐capped Keggin‐type GeNb_12_V unit with *C*_4v_ symmetry. The monovanadyl‐capped VO_5_ unit exhibits an axial V═O bond length of 1.640(40) Å and equatorial V─O bond lengths of 2.027(16) Å (Figure 1f). These bonds are slightly elongated compared to that in GeNb_12_V_2_ SBU, corresponding to the reduction of V^5+^ to V^4+^, which is consistent with the BVS calculations (Table S3, and Figures S6, S7). The SCSC transformation can be also confirmed by PXRD analyses of the bulk samples. As illustrated in Figure 2a, FZU‐3 and FZU‐3H feature similar PXRD peaks, with only minor peak shifts observed (Figure S8), indicating the retention of the space group *I*4/mmm. This observation aligns with the SXRD analysis and Fourier transformed infrared (FT‐IR) spectra (Figure S9).

Further tests were performed to elucidate the mechanism of the formic acid‐induced SCSC transformation. ICP‐OES data indicate that during the transformation process, a portion of the counter ions, namely Li^+^ and K^+^, is leached, while the V content remains unchanged. UV–vis spectroscopy (Figure 2b) reveals an increased absorption peak at approximately 700 nm in FZU‐3H, assigned to the d–d transition in V^4+^ (d^1^) indicating partial reduction of V^5+^ to V^4+^.^[^ ^34^ ^]^ This observation is corroborated by electron paramagnetic resonance (EPR) spectroscopy of FZU‐3H (Figure 2c), which shows a strong signal at *g* = 1.912, consistent with the presence of V^4+^ species. Elemental analysis confirms the absence of carbon atoms in FZU‐3H, thereby excluding the possibility that the EPR signal arises from formic acid radicals (Table S6). X‐ray photoelectron spectroscopy (XPS, Figure S10) further supports the presence of V^4+^, based on characteristic peaks at 517.5 eV and 516.4 eV corresponding to V^5+^ 2p_3/2_ and V^4+^ 2p_3/2_, respectively (Figure 2d); the increased intensity of the V^4+^ 2p_3/2_ peak in FZU‐3H indicates partial reduction of vanadium from V^5+^ to V^4+^.^[^ ^35^, ^36^ ^]^ X‐ray absorption spectroscopy (XAS) (Figure S11) demonstrates a shift in the V K‐edge absorption edge to lower energy in FZU‐3H compared to FZU‐3, further indicating the increase in V^4+^ content. Additionally, experiments involving formic acid‐induced SCSC transformations in the presence of Li^+^ yielded FZU‐3A and FZU‐3B (see Experimental Section in the Supporting Information). These findings suggest a plausible SCSC transformation mechanism wherein protons from dissociated formic acid leach monovalent counterions (Li^+^ and K^+^) via a proton concentration gradient, facilitating the detachment of high‐valent V^5+^ species into the interlayer, and formation of monovanadyl‐capped GeNb_12_V SBUs. Simultaneously, reaction of V^5+^ with formic acid leads to the formation of V^4+^ centers (Figures S12 and S13).

**Figure 3:**
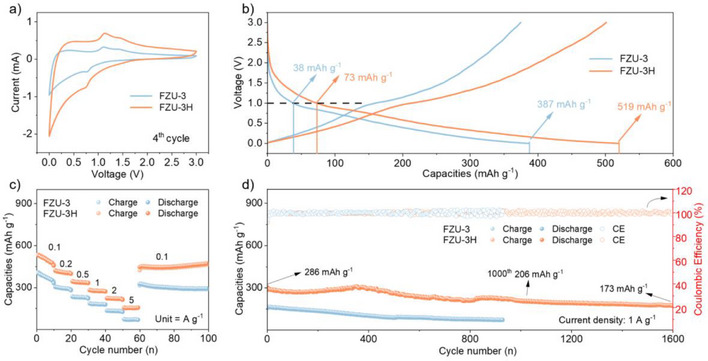
a) CV comparison of FZU‐3 electrode and FZU‐3H electrode at a scan rate of 1.0 mV s^−1^ in the voltage range 0.01–3.0 V (versus Li^+^/Li). b) Charge/discharge curve of FZU‐3 electrode and FZU‐3H electrode at 0.1 A g^−1^. c) Rate performance of FZU‐3 electrode and FZU‐3H electrode. d) Long‐term cycling performance of FZU‐3 electrode and FZU‐3H electrode at 1.0 A g^−1^.

To evaluate the influence of the SCSC transformation on the LIBs performance, we assembled 2032‐type coin cells using the two frameworks as anode materials. The cyclic voltammetry (CV) curves were first collected in the range of 0.01‐3 V (versus Li^+^/Li) at a scan rate of 1.0 mV s^−1^ (Figure S14). The initial negative scan exhibits a broad reduction peak that disappears in subsequent scans, which is attributed to the irreversible formation of solid‐electrolyte interphase (SEI).^[^ ^37^, ^38^ ^]^ Moreover, the overlapped CV curves in the following cycles indicate the formation of a stable SEI on the surface. XPS analysis before and after the first charge/discharge cycle (Figure S15) reveals the presence of F element after cycling, indicating that the SEI contains LiF. To analyze the differences between FZU‐3 and FZU‐3H, a comparison of their CV curves (the fourth cycle) is presented in Figure 3a, where a pair of similar reversible redox peaks at 1.7/0.83 V is distinctly observed, indicating that the overall structure of the crustal remains consistent after HCOOH immersion. Additionally, FZU‐3H displays a larger CV area compared to FZU‐3, indicating its higher capacity. The formation of the SEI film on the electrode is further corroborated by the charge/discharge curves (Figure S16), which show a low initial Coulombic efficiency (CE) of only 54%. Moreover, a discharge capacity of 676 mAh g^−1^ at a current density of 0.1 A g^−1^ is achieved for FZU‐3H in the second cycle, whereas FZU‐3 deliver only 473.0 mAh g^−1^. Figure 3b compares the charge/discharge profiles of FZU‐3 and FZU‐3H at a current density of 0.1 A g^−1^. FZU‐3 can deliver a capacity of 387 mAh g^−1^ (accommodated 33 Li^+^ ions), whereas after SCSC transformation, FZU‐3H demonstrates an enhanced capacity to 519 mAh g^−1^ (accommodated 42 Li^+^ ions), which are consistent with the results calculated from the CV curves at 0.2 mV s^−1^ (Figure S17). Furthermore, the discharge capacities primarily originate from the voltage region below 1.0 V. When discharged from 3 to 1 V, the FZU‐3 and FZU‐3H only deliver specific capacities of 38 and 73 mAh g^−1^, respectively. However, when discharged to 0.01 V, FZU‐3 and FZU‐3H achieved specific capacities of 387 and 519 mAh g^−1^, respectively.

The rate capability of FZU‐3H is also better than FZU‐3 (Figure 3c). The FZU‐3H electrode displays average reversible capacities of 480, 417, 345, 283, and 225 mAh g^−1^ at current densities of 0.1, 0.2, 0.5, 1, and 2 A g^−1^, respectively. Even at a higher current density of 5 A g^−1^, the FZU‐3H electrode still delivers a reversible capacity of 157 mAh g^−1^, significantly surpassing the FZU‐3 electrode (70 mAh g^−1^). Upon returning to the current density of 0.1 A g^−1^, the FZU‐3H electrode remains a capacity of 451 mAh g^−1^, demonstrating its excellent rate performance and stability. The improvement in rate performance is due to the faster Li‐ion insertion/extraction kinetics of the FZU‐3H. Moreover, the lower polarization voltage observed for the FZU‐3H electrode (Figure S18) corroborates its superior rate capability. Differential charge versus voltage curves across various cycles (Figure S19) reveal that the peak density of the FZU‐3H electrode decreases less, and its curves exhibit better similarity and overlap compared to FZU‐3. This indicates that FZU‐3H experiences a smaller loss in capacity per unit voltage and less active material loss during cycling.^[^ ^39^, ^40^ ^]^ Additionally, the smaller peak shift observed for FZU‐3H suggests lower resistance to Li^+^ ions insertion and extraction during the cycling process.^[^ ^41^ ^]^ To demonstrate the advantages of FZU‐3H electrode, we investigated the capacity contributions of the two frameworks (Figure S20). The FZU‐3H electrode exhibits a reversible capacity of 738 mAh g^−1^ after 400 cycles at a current density of 0.1 A g^−1^, which is much higher than that of FZU‐3 electrode of 343 mAh g^−1^.

Long‐term stability is a crucial parameter for anode materials in practical applications. As shown in Figure 3d, the FZU‐3H electrode retains reversible capacities of 206 and 173 mAh g^−1^ after 1000 cycles and 1600 cycles, respectively, at a high current density of 1 A g^−1^ with a capacity loss rate of only 0.025% per cycle. In sharp contrast, the FZU‐3 electrode exhibits poor cycling stability with a capacity loss rate of 0.061% per cycle. The superior performance of FZU‐3H is further supported by comparison with state‐of‐the‐art niobium‐based and POM‐based anode material reported previously (Figure S21 and Tables S7, S8), where FZU‐3H shows higher specific capacity and longer lifespan compared with the reported samples. This suggests that FZU‐3H is a highly promising candidate for fast‐charging, durable battery electrodes based on niobate frameworks.

**Figure 4:**
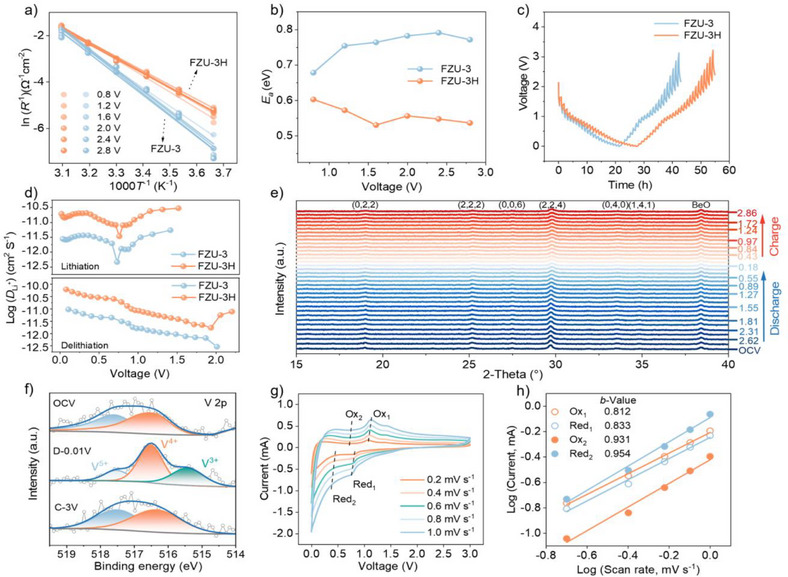
a) Arrhenius plots showing the proportional relation between logarithmic values of the reciprocal of charge‐transfer resistance and the reciprocal of the absolute temperatures for 2.8, 2.4, 2.0, 1.6, 1.2, and 0.8 V. b) Activation‐energy profiles for FZU‐3 and FZU‐3H electrodes at various potentials. c, d) GITT curves and corresponding Li^+^ diffusion coefficients of FZU‐3 and FZU‐3H electrode at 0.05 A g^−1^ after the sixth cycle. e) In situ XRD patterns of FZU‐3H electrodes at different charge/discharge stages in the first cycle. f) V 2p XPS spectra of FZU‐3H electrode at different charge/discharge stages in the first cycle. g) CV curves of FZU‐3H electrode at different scan rates. h) Plots of peak currents and logarithmic scan rates of FZU‐3H electrode.

To gain a deeper insight into the superior electrochemical storage performance of the FZU‐3H electrode compared to that of FZU‐3, we measured the charge transfer resistance (*R_ct_*) of the electrode at the corresponding potential under different temperatures. This approach allows the determination of the activation energy (*E*_a_) for the redox reaction process. Therefore, electrochemical impedance spectroscopy (EIS) measurements are performed from 0.8–2.8 V under different temperatures (273 K, 283 K, 293 K, 303 K, 313 K, 323 K) in a frequency range from 0.1 Hz to 100 kHz (Figure S22 and S23). An equivalent circuit model is employed to fit the impedance spectrum (Figure S24). According to the Arrhenius equation, the reciprocal of the *R_ct_* is linearly related to the reciprocal of the absolute temperature. By fitting charge‐transfer impedances measured at different temperatures, the *E*_a_ at different potentials can be obtained by the slope of the linear fitting (Figure 4a). It can be observed that the *E*_a_ values of FZU‐3H are smaller than those of FZU‐3 at 0.8–2.8 V (Figure 4b). The maximum *E*_a_ of electrode decreases from 0.79 eV in FZU‐3 to 0.55 eV in FZU‐3H. This indicates that the FZU‐3H electrode is thermodynamically more favorable than the FZU‐3, facilitating lithiation/delithiation more effectively.^[^ ^42^, ^43^ ^]^

The galvanostatic intermittent titration technique (GITT)^[^ ^44^ ^]^ was employed to monitor the electrochemical reaction kinetics of the two electrodes during different lithiation/delithiation stages (Figure 4c). It can be observed that the Li^+^ diffusion coefficients in FZU‐3 range from 5.51 × 10^−12^ to 2.96 × 10^−13^ cm^2^ S^−1^ during the lithiation/delithiation process, whereas in FZU‐3H, the values are significantly higher, ranging from 3.01 × 10^−11^ to 1.72 × 10^−12^ cm^2^ S^−1^ (Figure 4d). The FZU‐3H electrode exhibits smaller overpotentials and higher Li^+^ diffusion coefficients than the FZU‐3 electrode for both Li^+^ insertion and extraction processes, suggesting improved Li^+^ diffusion kinetics, which enhances the rate performance of FZU‐3H. According to the fitting results of the Nyquist plots, the *R*_ct_ values of FZU‐3 and FZU‐3H are 173.6 and 47.7 Ω, respectively (Figure S25). The lower *R*_ct_ value indicates a faster charge transfer of FZU‐3H. Moreover, after 1000 cycles, the *R*_ct_ of FZU‐3H electrode shows the only minimal changes (Figure S26), indicating the superior structural stability of FZU‐3H. XRD before and after cycling also further demonstrated the structural stability of FZU‐3H (Figure S27).

To investigate the reaction mechanism of FZU‐3H electrode for lithium storage, in situ PXRD measurements were performed. As shown in Figure 4e, the diffraction peaks of the FZU‐3H observed in the diffractograms remain almost unchanged during the continuous discharge process, indicating that the discharging process primarily involves Li^+^ intercalation without significant structural transformation. When continuously charging to 3 V, the diffraction peaks of FZU‐3H remain negligible changes with the gradual increase of voltage, demonstrating that the FZU‐3H exhibits outstanding structural stability. The evolution of the elemental valence state can be investigated via XPS characterization (Figure 4f). The original FZU‐3H electrode contains vanadium in oxidation states +4 and +5, based on analysis of their binding energies located at 516.5 (V^4+^ 2p_3/2_) and 517.5 eV (V^5+^ 2p_3/2_).^[^ ^45^, ^46^ ^]^ After discharging to 0.01 V, a new peak corresponding to V^3+^ (V^3+^ 2p_3/2_ at 515.4 eV) can be observed, indicating the reduction of vanadium upon discharge. Upon fully charging to 3 V, the V^3+^ peak disappears, while the V^4+^/V^5+^ signals reappear, indicating the reversibility of the vanadium redox processes. This result indicates that each V center in FZU‐3H electrode is generally able to accommodate 1–2 Li^+^ ions (12.33–24.66 mAh g^−1^). The Nb 3d core level spectra were also collected (Figure S28), showing two peaks at −209.6 and −206.9 eV relating to 3d_5/2_ and 3d_3/2_ for Nb^5+^.^[^ ^47^, ^48^ ^]^ During the discharge/charging process, the peak for Nb^5+^ was essentially unchanged, further demonstrating the stability of its structure. These observations verify the outstanding structural stability of FZU‐3H electrode during the reversible Li^+^ insertion/extraction.

To further understand the factors contributing to the fast Li^+^ storage ability of FZU‐3H electrode, kinetic studies were performed using CV curves recorded at scan rates ranging from 0.2 to 1.0 mV s^−1^ (Figure 4g and S29a). The peak current (*i*) is plotted logarithmically against the scan rate (*v*), following the relationship of *i* = *a*·*ν^b^*, where *a* is a constant, and the *b*‐value is derived from the slope of log (*i*) versus log (*v*) plot. A *b*‐value of 0.5 indicates a diffusion‐controlled mechanism, while a *b*‐value of 1.0 suggests capacitive behavior.^[^ ^49^, ^50^ ^]^ The *b*‐values of the two redox peaks of Ox_1_ and Red_1_ for both electrodes are determined to fall between 0.5 and 1.0 V (Figures 4g and S29b), indicating that the charge storage mechanism involves a combination of ion diffusion and surface capacitance processes. Moreover, the *b*‐values of 0.812 and 0.833 for FZU‐3H electrode are higher than those for FZU‐3 electrode (0.705 and 0.713), suggesting faster reaction kinetics for FZU‐3H electrode. Interestingly, the *b*‐value in the voltage range of 0.01‐1 V is approximately equal to 1 (Figure 4h), implying purely capacitive control in this voltage region. The capacitance and diffusion contribution curves presented in Figure S30 shows that the capacitance contribution increases with higher scan rates. As the scan rate increases from 0.2 to 1.0 mV s^−1^, the capacitance contribution of the FZU‐3H electrode rises from 49% to 69%, notably higher than FZU‐3, indicating that FZU‐3H is more favorable for surface pseudocapacitive charge storage. These results further demonstrate that FZU‐3H exhibits superior reaction kinetics compared to FZU‐3. Furthermore, solid‐state ^7^Li nuclear magnetic resonance (NMR) spectroscopy shows that the full width at half‐maximum (FWHM) of the ^7^Li NMR signal decreased significantly when comparing FZU‐3 (4.47 kHz) to FZU‐3H (0.93 kHz), see Figure S31. This indicates a longer spin–spin relaxation time (*T*₂) associated with rapid Li⁺ motion within FZU‑3H.^[^ ^51^, ^52^ ^]^

**Figure 5:**
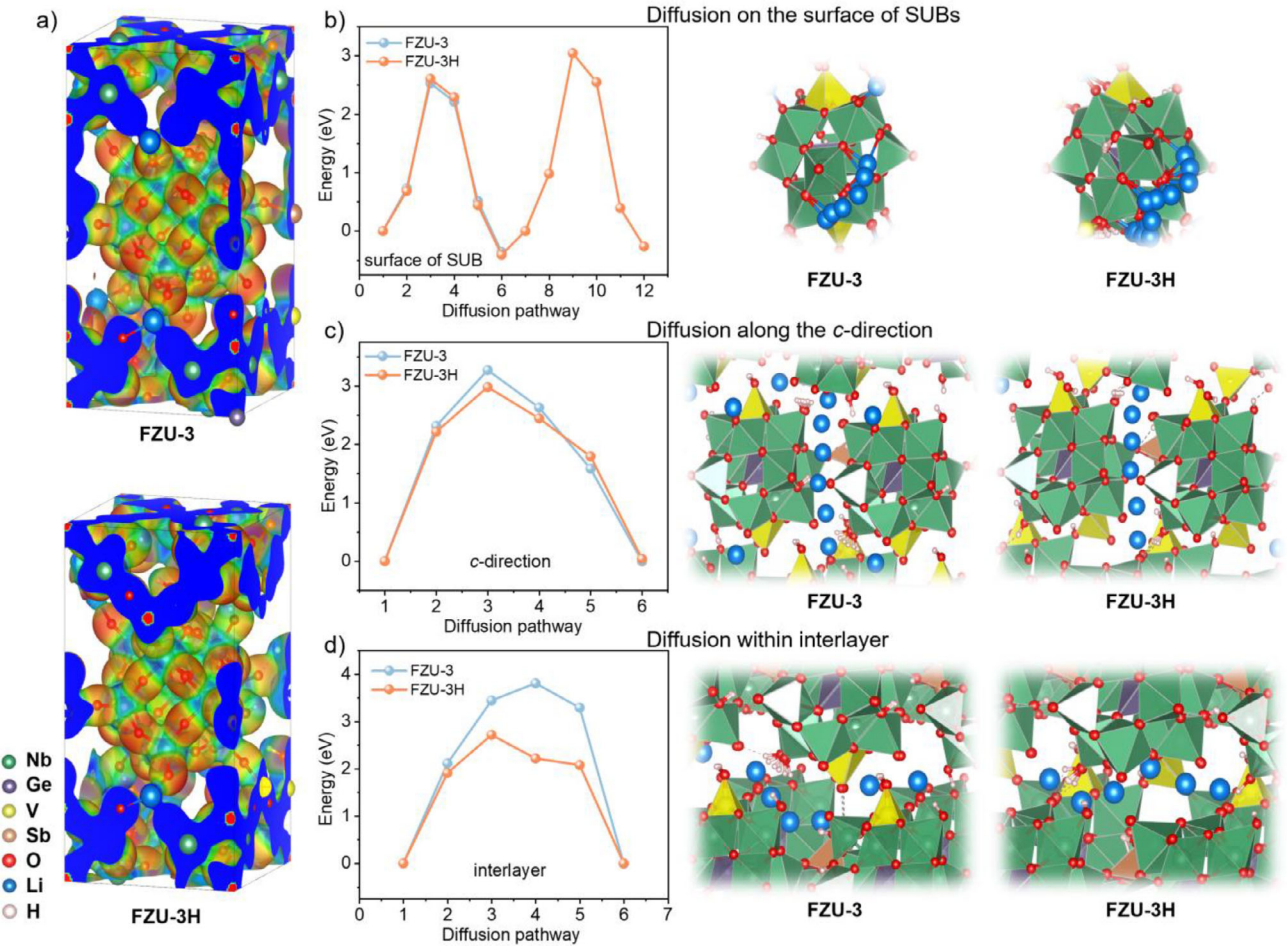
a) Electrostatic potential of FZU‐3 and FZU‐3H with isosurfaces of 0.02 e/Bohr^3^. Red and blue indicate regions of low and high electrostatic potential, respectively. b)–d) Simulated Li diffusion energy barrier and pathway of in the surface of SBUs, c‐direction and interlayer.

To obtain the fundamental insights into the better anode performance after SCSC transformation, density functional theory (DFT) simulations are performed. A key influence on the performance of anode materials is the storage capacity, which is closely affected by the number of Li^+^ binding sites and the size of the Li^+^ storage space within the materials. The electrostatic potential calculations (Figure 5a) reveal that the bridged oxo atoms in the SBUs exhibit negative charge, enabling the vacancy sites in the three‐ring and four‐ring window to adsorb Li^+^ ions, consistent with reported findings.^[^ ^53^ ^]^ During the HCOOH‐induced SCSC transformation, the V═O units originally bound to the four‐ring window sites of the bivanadyl‐capped GeNb_12_V_2_ SBUs dissociate and migrate, forming monovanadyl‐capped GeNb_12_V SBUs. This transformation exposes more four‐ring window sites capable of binding more Li^+^ ions, thereby enhancing the lithium capacity, which can also be verified by the higher specific surface area of FZU‐3H (Figure S32). In addition, another crucial characteristic of anode materials is their charge/discharge rate, which is heavily influenced by the mobility of Li⁺ ions. The mobility of Li^+^ ions depends on the diffusion barrier, with lower diffusion barriers facilitating faster migration. The diffusion barrier calculations indicate that the diffusion barriers for Li^+^ ions on the surface of SBUs (Figure 5b) and along the *c*‐direction between two SBUs (Figure 5c) remain nearly unchanged before and after the SCSC transformation. However, the interlayer diffusion energy barrier for Li^+^ ions in FZU‐3H is significantly lower than that in FZU‐3 (Figure 5d). This result suggests that the SCSC structural transformation primarily enhances the migration velocity of interlayer Li^+^ ions, thereby further improving the charge/discharge rate performance.

## Conclusion

In summary, we have successfully developed a rare all‐inorganic 2D PONb framework with SCSC transformation capability, demonstrating its potential as an advanced anode material for LIBs. Driven by the SCSC transformation and simultaneous structural and redox‐changes at the vanadium centres, FZU‐3H exhibits faster reaction kinetics and an enhanced Li⁺ ion diffusion rate. As a result, FZU‐3H achieves a specific capacity of 571 mAh g^−1^ at a current density of 0.1 A g^−1^, making this one of the best‐performing niobate materials for LIB electrodes. Electrochemical evaluations and in situ XRD results revealed that the high capacity and fast kinetics of FZU‐3H are primarily attributed to capacitance within the low voltage regime. Moreover, theoretical calculations indicated that the SCSC transformation created more voids, allowing for the accommodation of more Li^+^ ions, and lowers the Li^+^ ions transfer energy barriers. Our work introduces a novel PONb with well‐defined structure, providing a new design strategy for LIBs anode aimed at practical applications.

## Author Contributions

Cai Sun: Writing, Data analysis & Software. Jian‐Ping Chen: Synthesis & Original Draft. Yan‐Lan Wu: Synthesis & Characterization. Yi‐Ying Li: Validation. Xin‐Xiong Li: Structural analysis. Ping‐Wei Cai: Conceptualization, Review, Electrochemical research & Funding acquisition. Carsten Streb: Conceptualization, Writing, Review & Editing. Shou‐Tian Zheng: Project administration, Conceptualization, Review, Editing, and Funding acquisition.

## Conflict of Interests

The authors declare no conflict of interest.

## Supporting information



Supporting Information

## Data Availability

The data that support the findings of this study are available from the corresponding author upon reasonable request.
