# Supplementary material for: Single‐Crystal‐to‐Single‐Crystal Transformation in a Thermally Stable All‐Inorganic Polyoxoniobate Framework Boosts Lithium Ion Battery Anode Performance
Source: Angew Chem Int Ed Engl. 2025 Jun 3;64(31):e202506533. doi: 10.1002/anie.202506533 (PMC12304815; doi:10.1002/anie.202506533)
Supplement: Supplementary file 1 — Supporting Information [file ANIE-64-e202506533-s001.pdf]

Supporting Information
©Wiley-VCH 2025
69451 Weinheim, Germany

Single-Crystal-to-Single-Crystal Transformation in a Thermally Stable All-Inorganic Polyoxoniobate Framework Boosts Lithium Ion Battery Anode Performance

Cai Sun,[a+] Jian-Ping Chen,[a+] Yan-Lan Wu,[a+] Yi-Ying Li,[a] Xin-Xiong Li,[a] Ping-Wei Cai,[a*] Carsten Streb,[b*] Shou-Tian Zheng[a*]

[a] Prof. Dr. C Sun, J.-P. Chen, Dr. Y.-L. Wu, Y.-Y. Li, Prof. Dr. X.-X. Li, Prof. Dr. P.-W. Cai, Prof. Dr. S.-T. Zheng
Fujian Provincial Key Laboratory of Advanced Inorganic Oxygenated-Materials
College of Chemistry
Fuzhou University
Fuzhou, Fujian 350108, China
E-mail: cai2022@fzu.edu.cn; stzheng@fzu.edu.cn

[b] Prof. Dr. C Streb
Department of Chemistry
Johannes Gutenberg University Mainz
Duesbergweg 10-14 55128 Mainz, Germany
E-mail: carsten.streb@uni-mainz.de

[+] These authors contribute equally to this work.

Experimental Procedures

1. Materials

$K_7H[Nb_6O_{19}]\cdot 13H_2O$ precursor was synthesized as described in the literature.[1] Other chemicals were used as purchased without further purification. The concentration of HCOOH used was 88 wt% (Sinopharm Chemical Reagent Co., Ltd).

2. Measurements

Infrared (IR) spectra (KBr pellet) were performed on an Opus Vetex 70 FT-IR infrared spectrophotometer in the range of 400–4000 cm^{-1}. UV-vis spectra were performed on a SHIMADZU UV-2600 UV-visible spectrophotometer by using the $BaSO_4$ as the blank. Powder X-ray diffraction (PXRD) patterns were recorded on an Ultima IV diffractometer with Cu-*K*α radiation (λ = 1.54056 Å) in the range of 5-50°. Simulated PXRD pattern was derived from the Mercury Version 4.3.0 software using the X-ray single crystal diffraction data. Inductively coupled plasma analyses were conducted on an Ultima2 Spectrometer (ICP-OES). Thermogravimetric analyses were conducted using a Mettler Toledo TGA/SDTA 851e analyzer in an N_2-flow atmosphere with a heating rate of 10 °C/min at a temperature of 30-1000 °C. XPS analyses were conducted on a Thermo Scientific K-Alpha with an operating voltage of 12 kV and a filament current of 6 mA. Note: XPS is a surface analysis technique with a probing depth of 1 to 10 nm, which offer insights into the bulk elemental composition and chemical states to a certain extent.[2] Optical images of the samples were obtained by iPhone 14 Pro from a fixed position under constant lighting conditions. The pH values of all solutions were determined by Mettler Toledo FiveEasy Plus. The content of the elements C,H,N in the samples was determined by CHN elemental analysis (EA, Vario EL Cube). X-ray absorption spectroscopy (XAS) data of V K-edge were performed on an easyXAFS 300+. The solid-state ^7Li NMR measurements were performed on a Bruker AVANCE NEO 400 M spectrometer (B_0 = 9.4 T, Larmor frequency ω_0 = 155.5 MHz for ^7Li) at room temperature. N_2 adsorption/desorption measurements were conducted on the ASAP 2020.

3. Synthesis.

$K_5Li_2Sb(H_2O)(GeNb_{12}O_{40}(V^{(V)}O)_2)$ $8H_2O$ (FZU-3): A mixture of $K_7HNb_6O_{19}\cdot 13H_2O$ (0.280 g, 0.204 mmol), V_2O_5 (0.045 g, 0.247 mmol), Sb_2O_3 (0.109 g, 0.373 mmol), GeO_2 (0.020 g, 0.191 mmol), LiCl (0.060g, 1.415 mmol), 200 µL en was mixed in 6 mL of H_2O. After stirring for 1 hour to get a pale yellow solution, the resulting mixture was sealed in a Teflon-lined autoclave (23 mL) and heated at 160 °C for 5 days. After cooling down, plae-brown block crystals were obtained. Yield: 145 mg (55.5%, based on Nb). The pH values before and after reaction were ca. 13.0 and 12.5, respectively. IR (KBr, cm^{-1}): 3290(m), 1619(s), 971(s), 861(s), 765(w), 620(m), 577(w), 533(m), 475(s), 431(w), 417(w).

$H_{1.5}K_2LiV^{(V)}_{1.5}Sb(H_2O)[GeNb_{12}O_{40}(V^{(IV)}O)_{0.5}]\cdot 8H_2O$ (FZU-3H): The FZU-3H was obtained by soaking FZU-3 crystals in HCOOH for 5 minutes. Afterwards, the residual HCOOH was removed from the surface of the crystals by rinsing several times with distilled water, resulting in the formation of yellowish-green block crystals. IR (KBr, cm^{-1}): 3522(m), 3238(m), 1641(s), 1576(s), 1364(m), 1160(w), 1101(w), 1036(s), 912(s), 831(s), 722(w), 598(s), 504(s), 423(w).

$H_{3-x}K_4Li_2Sb(H_2O)[GeNb_{12}O_{40}(V^{(V)}O)_x(V^{(IV)}O)_{2-x}]\cdot 8H_2O$ (0<x<2) (FZU-3A): The FZU-3A was obtained by soaking FZU-3 crystals in 10mL of 0.1 mol/L LiCl (1 mmol) and 20µL (0.029 mmol) of HCOOH for 15 minutes. Afterwards, the residual HCOOH was removed from the surface of the crystals by rinsing several times with distilled water, resulting in the formation of yellowish- green block crystals.

$H_{2.6-x-y}K_3Li_{1.4}(V^{(V)})_xSb(H_2O)[GeNb_{12}O_{39+x}(V^{(V)}O)_y(V^{(IV)}O)_{2-x-y}]\cdot 8H_2O$ (x+y<2; x, y>0) (FZU-3B): The FZU-3B was obtained by soaking FZU-3 crystals in 10mL of 0.1 mol/L LiCl (1 mmol) and 450µL (0.67 mmol) of HCOOH for 7 minutes. Afterwards, the residual HCOOH was removed from the surface of the crystals by rinsing several times with distilled water, resulting in the formation of yellowish-green block crystals.

Note: Due to the low molar quantity of reduced V in FZU-3A and FZU-3B, which cannot be accurately determined, the number of protons are determined by the equilibrium of electricity prices, while the contents of the other elements are determined with the aid of ICP ananlysis.

4. Elemental analysis

FZU-3, Calcd: Nb, 45.4%, Ge, 2.9%, Sb, 4.9%, V, 4.1%, K, 7.9%, Li, 0.56%; Found: Nb 45.4%, Ge, 3.0%, Sb, 10.6%, V, 3.6%, K, 8.92%, Li, 0.89%. FZU-3H, Calcd: Nb, 48.3%, Ge, 3.1%, Sb, 5.3%, V, 4.4%, K, 3.4%, Li, 0.30%; Found: Nb 44.6%, Ge, 3.0%, Sb, 8.9%, V, 3.6%, K, 3.4%, Li, 0.27%. FZU-3A, Calcd: Nb, 46.1%, Ge, 3.0%, Sb, 5.0%, V, 4.2%, K, 6.4%, Li, 0.57%; Found: Nb 41.3%, Ge, 2.7%, Sb, 9.0%, V, 3.6%, K, 5.4%, Li, 0.56%. FZU-3B, Calcd: Nb, 47.2%, Ge, 3.1%, Sb, 5.2%, V, 4.3%, K, 5.0%, Li, 0.41%; Found: Nb 41.3%, Ge, 2.7%, Sb, 9.0%, V, 3.8%, K, 4.3%, Li, 0.36%.

Note: The crystal samples were vacuum dried at 60 ℃ for one day before ICP testing, and the calcd. values did not consider the contribution of crystal water. Because the optimal emission wavelength for Sb (206.833 nm) overlaps with that of Ge (206.866 nm), the ICP-OES test results show an Sb concentration that is twice the theoretical value.

5. X-ray crystallography

Single-crystal X-ray diffraction measurements were performed on a Bruker APEX II diffractometer, using graphite monochromated Mo K_α radiation (λ = 0.71073 Å) at 175(2) K. Intensity data sets were collected using ω scan techniques, and corrected for *Lp* effects. The structures were solved by direct method and refined by full-matrix least squares on F^2 using the Siemens SHELXTL^{TM} Version 5 package of crystallographic software with anisotropic thermal parameters for all atoms. The entries of CCDC 2408261, 2408262,

2428643 and 2429554 contain the supplementary crystallographic data for FZU-3, FZU-3H FZU-3A and FZU-3B. These data can be obtained free of charge at http://www.ccdc.cam.ac.uk/conts/retrieving. html or from the Cambridge Crystallographic Data Centre, 12, Uni on Road, Cambridge CB2 1EZ, U.K. Fax: (Internet) +44-1223/336-033. E-mail: depos-it@ccdc.cam.ac.uk. All the data supporting the findings of this study are available within the article and its Supplementary Information and also from the corresponding authors upon reasonable request.

The bond valences of all elements are calculated by the formula $S_{ij} = \exp[(R_{ij}-R_0)/B]$, where R is the bond length and R_0 (the length of a bond of unit valence), B are fitting constants. The parameter of Ge reported from Caminiti;[3] Sb from Brown;[4] V and Nb from Tytko.[5]

The approach to handling the vanadium cap site is shown as follows:

The vanadium cap site of FZU-3H occupies a special position with an occupancy factor of 0.125. Through free refinement, we found that the occupancy factor of the vanadium cap site is 0.02943, corresponding to approximately 0.24 V atoms (0.02943/0.125) at this site, with the isotropic displacement parameter U(iso) reduced to 0.03. To achieve an integer or fractional stoichiometric ratio in the chemical formula, we constrained the occupancy factor of the vanadium cap site to 0.03125, equivalent to 0.25 V atoms at this site, with a corresponding U(iso) value of 0.036. Consequently, after reduction with formic acid, only 0.5 V atoms (there are two cap sites per cluster, i.e., 0.25 × 2 = 0.5) remained on the bivanadyl-capped SBU $\{PV_2Nb_{12}O_{42}\}$, indicating that 1.5 V atoms had moved away from their original cap sites. Bond valence sum (BVS) calculations revealed that the oxidation state of the 0.5 V cap atoms remaining on the cluster was +4.

Treatment of the disordered metal sites and residual electron density:

ICP-OES indicates that the initial structure FZU-3 contains approximately two equivalents of Li^+ ions per formula unit. Due to the presence of significant residual electron density peaks at the tetrahedral sites, which align with the coordination environment expected for lithium ions, it is plausible that lithium ions occupy these positions. However, assigning lithium ions to these sites results in unreasonably high thermal parameters, suggesting positional disorder. This disorder may be indicative of the lithium-ion mobility within the structure.

To further assess the mobility of Li ions, solid-state ^{7}Li NMR spectroscopy was performed using natural abundance LiCl as a reference. As shown in the ^{7}Li MAS-NMR spectra, strong resonance bands corresponding to the central transition (CT) of ^{7}Li (I = 3/2) were observed at approximately –1.08 ppm (for FZU-3) and –5.25 ppm (for FZU-3H). This observation supports the formic acid induced changes in the chemical environment of the Li ions, and is indicative of the mobility of the Li ions. Furthermore, after the formic acid-induced single-crystal-to-single-crystal transformation, the full width at half-maximum (FWHM) of the ^{7}Li NMR signal decreased significantly from 4.47 kHz to 0.93 kHz, implying an increased spin–spin relaxation time (T_2) which suggests rapid Li^+ motion within FZU-3H (for related examples of Li ion mobility, see *Nat. Commun.* **2023**, *14*, 3780; *Angew. Chem. Int. Ed.* **2024**, *63*, e202317949). This is consistent with the findings from the electrochemical impedance spectroscopy tests (Figure 4a) and the density functional theory calculations of the Li-ion migration barrier (Figure 5) presented in the manuscript.

Formic acid-induced single-crystal-to-single-crystal transformation gave compound FZU-3H, where the electron density at the cap sites for vanadium decreased, while a significant, diffuse residual electron density was observed at the tetrahedral sites. Based on ICP-OES, leaching of Li^+ and K^+ ions was observed while the overall vanadium content remained unchanged. This suggests that during the single-crystal-to-single-crystal conversion, a minor amount of the vanadium detach from the cap sites and may migrate to the tetrahedral sites. These findings indicate that both Li^+ and V^{5+} ions partially occupy the tetrahedral sites; however, accurately quantifying their respective occupancies is difficult due to disorder and nonstoichiometric distribution within the bulk. Thus, the revised crystallographic data (CIF files), we have retained the residual electron density at the tetrahedral positions. Retaining residual electron density assigned to disordered counter-cations is a common practice in polyoxometalates (*Angew. Chem. Int. Ed.* **2021**, *60*, 12461-12466；*J. Am. Chem. Soc.* **2019**, *141*, 13479-13486; *J. Am. Chem. Soc.* **2019**, *141*, 19550-19554；*J. Am. Chem. Soc.* **2018**, *140*, 2595-2601). The number of non-hydrogen counter-cations in the molecular formula were determined by ICP-OES analysis.

Notably, electrochemical impedance spectroscopy and ion migration activation energy calculations reveal that FZU-3H exhibits enhanced specific capacity and charge–discharge rates, attributed to the loss of capped V atoms, which facilitates increased Li^+ mobility. Therefore, the specific identity of the ions occupying the tetrahedral sites is not the primary factor influencing the electrochemical performance of the lithium battery.

6. Electrochemical Characterization

6.1 Anode preparation

The anode was fabricated by mixing active materials (70 wt%), acetylene black (20 wt%), and sodium carboxymethyl cellulose (10 wt%) into a uniform slurry, followed by coating onto a copper foil. The pellets were dried in a vacuum at 60 °C for 24 h. The mass loading of the anode active material on each electrode (diameter: 12 mm) was ~1.2 mg cm^{-2}.

6.2 Electrochemical measurements

The electrochemical measurements were investigated with 2032-type coin cells assembled in a glove box filled with argon atmosphere (H_2O < 0.5 ppm, O_2 < 0.5 ppm). For LIBs, the Li metal foils as the counter/reference electrode, 1 M $LiPF_6$ dissolved in diethyl carbonate (DEC)/ethylene carbonate (EC)/methyl ethyl carbonate (MEC) (2:3:5 by wt%) as electrolyte and PP membrane as the separator. Then, the assembled cells were aged for 20 h to ensure that the system was fully wetted. The cell cycling experiments are carried out in a climatic chamber with a constant temperature of 35 °C. The electrochemical performances of the batteries were tested on a Land battery testing system (LANDTE Co., China) with a potential window of 0.01–3 V for SIBs, and CV tests were measured on a CHI660E electrochemical station (CHI Instrument Co., Shanghai, China), respectively. For the long-term cycling performance at high specific currents, the LIBs were first activated at 0.1 A g^{-1} for ten cycles and then were operated at a higher

specific current for a long-cycle test. An AC voltage amplitude of open-circuit voltage was employed to measure EIS within the frequency range from 0.01 Hz to 100 kHz. The specific capacity and specific currents were calculated based on the weight of anode materials. The galvanostatic intermittent titration technique (GITT) tests were measured under constant-current conditions at 0.05 A g^{-1} for 20 min and rest intervals for 40 min after the 10th cycle.

6.3 Activation energy of redox reaction

The LIBs were assembled as described above and were initially discharged and charged at 1 A g^{-1} for 5 cycles prior to the EIS tests. Subsequently, the cells were placed in a thermostatic chamber set to specific temperatures (273, 283, 293, 303, 313, and 323 K) for 1 hour. Impedance spectra were then measured at various potentials ranging from 0.2 V to 2.8 V using the same electrode under the specified temperature. Finally, the activation energy of the redox reaction was calculated at different potentials.

6.4 Calculation of b-value

The electrochemical kinetics within the cell were investigated using cyclic voltammetry (CV) at various scan rates. The measured current from the CV curves can be attributed to two distinct charge-storage mechanisms: the diffusion-controlled faradaic contribution and the surface capacitive-dominated processes. Generally, the relationship between peak current (*i*) and scan rate (*v*) can be described using the following formulas:[8]

$$i = a\, v^{b}$$

$$log(i) = b\ log(v) + log(a)$$

Where a and b are the adjustable parameters. In the context of the redox process of the electrode, the b-value indicates the nature of the charge storage mechanism. Specifically, when b approaches 1.0, it suggests an ideally capacitive contribution process. On the other hand, when b approaches 0.5, it indicates a predominantly faradaic intercalation process.

6.5 Calculation of capacitive contribution

The ratios of Li^{+} capacitive contribution can be further quantitatively distinguished by separating the current response (*i*) at a specific potential (*V*) according to the following equations:[9]

$$i(V) = k_1 v + k_2 v^{1/2}$$

$$i(V)/v^{1/2} = k_1 v^{1/2} + k_2$$

Where both k_1 and k_2 are constant values obtained from the slope and intercept of the $i(V)/v^{1/2}$ versus $v^{1/2}$ plot, respectively. And k_1v and $k_2v_{1/2}$ represent the capacitive contribution and diffusion contribution, respectively.

6.6 Calculation of Calculation of D_{Li^+} value

The Li ions diffusion coefficient (D_{Li^+}) can be calculated from the GITT potential profiles according to the simplified Fick's second law with the following equation:[10]

$$D = \frac{4}{\pi\tau}\left(\frac{m_B V_M}{M_B S}\right)^2\left(\frac{\Delta E_s}{\Delta E_\tau}\right)^2$$

Where m_B is the electrode active mass and *S* (cm^2) is the geometric area. ΔE_s is the quasi-thermodynamic equilibrium potential difference between before and after the current pulse. ΔE_τ is the duration of the current pulse and the change of voltage during the current pulse. M_B (g mol^{-1}) is the molecular weight and V_M (cm^3 mol^{-1}) is the molar volume of electrode material.

7. Computational approaches

First-principles DFT simulations were performed using the VASP package. The electron exchange-correlation (XC) energies were treated by the generalized gradient approximation (GGA) in the Perdew Burke Ernzerhof (PBE) scheme.[11] In addition, the projection-enhanced wave (PAW) pseudopotential was used to describe the ion nucleus and valence electron interactions.[12] A kinetic energy cutoff of 400 eV was chosen as the plane wave basis set. The crystal structure of FZU-3 and FZU-3H were applied to build calculation models. Since we are only interested in the interaction behavior between Li^{+} ions and the framework, all K^{+} ions were removed for simplicity, and protons were randomly added to the framework to balance the charge. For FZU-3 and FZU-3H, where structural units like GeO_4 polyhedra, Sb–O linkers, and monovanadyl {V=O} are positionally disordered, one specific configuration was selected for the calculations. All structural models were fully optimized until the force was less than 10^{-2} eV $Å^{-1}$ and the energy converged to 10^{-5} eV between two consecutive self-consistent steps. The integration over the reciprocal space was performed using a Monkhorst model with a *Γ*-point. The migration behavior of Li in different regions of the model was evaluated using the climbing-image NEB method.[13] Due to the large number of atoms in the framework and the significant computational cost of searching for ion diffusion paths in three-dimensional space, we simplified the calculations by allowing the atoms surrounding the diffusion path to move freely while freezing the rest, to qualitatively evaluate the diffusion energy barrier. A 1×2×1 supercell was constructed to better represent Li^{+} diffusion along the *c*-direction and interlayer pathways.

Supplementary data

Table S1. Crystal and structure refinement data for FZU-3, FZU-3H, FZU-3A and FZU-3B.

	**FZU-3**	**FZU-3H**	**FZU-3A**	**FZU-3B**
**Formula**	$H_{18}Li_2K_5SbGeNb_{12}V^{(V)}{}_2O_{51}$	$H_{19.5}LiK_2SbGeNb_{12}V^{(IV)}{}_{0.5}V^{(V)}{}_{1.5}O_{49.5}$	$H_{21-x}Li_2K_4SbGeNb_{12}V^{(IV)}{}_{2-x}V^{(V)}{}_xO_{51}$	$H_{20.6-x-y}K_3Li_{1.4}SbGeNb_{12}V^{(IV)}{}_{2-x-y}V^{(V)}{}_{x+y}O_{50}$
Mr	2454.6	2307.9	2418.6-x	2358.9-x-y
**Crystal system**	Tetragonal	Tetragonal	Tetragonal	Tetragonal
**Space group**	*I*4/mmm	*I*4mmm	*I*4mmm	*I*4mmm
a **(Å)**	10.77(5)	10.69(2)	10.72(4)	10.68(3)
b **(Å)**	10.77(5)	10.69(2)	10.72(4)	10.68(3)
c **(Å)**	19.99(10)	19.76(7)	20.05(1)	19.87(1)
α **(°)**	90	90	90	90
β **(°)**	90	90	90	90
γ **(°)**	90	90	90	90
V **($Å^3$)**	2318.6(2)	2260.06(11)	2305.1(2)	2267.25(18)
D_{calcd} **(g/cm^3)**	3.376	3.158	3.258	3.175
Z	2	2	2	2
F**(000)**	2182	1985	2087	2000
**Reflns collcd/unique (R_{int})**	5319/632 (0.0378)	6601/741 (0.0288)	6195/728 (0.0198)	6599/793 (0.0189)
**Data/params/restraints**	632/0/69	741/0/73	728/30/74	793/6/73
$R_1{}^a$, $\omega R_2{}^b$ **[I>2σ]**	0.0623, 0.1764	0.0739, 0.2252	0.0816, 0.2317	0.0788, 0.2414
$R_1{}^a$, $\omega R_2{}^b$ **[all data]**	0.0697, 0.1847	0.0940, 0.2398	0.0873, 0.2379	0.0842, 0.2483
**GOF on F^2**	1.131	1.106	1.116	1.120
$\Delta\rho_{max}$ **and** $\Delta\rho_{min}$ **($e/Å^3$)**	2.77/-2.40	4.63/-1.23	4.85/-3.85	5.55/-1.42

$^aR_1 = \sum||F_o|-|F_c||/\sum|F_o|$, $^b\omega R_2 = \{\sum\omega[(F_o)^2-(F_c)^2]^2/\sum\omega[(F_o)_2]^2\}^{1/2}$.

Table S2. Bond valence summations of metal atoms of FZU-3.

Atom1	Atom2	R_{ij}	R_{o}	B	S_{ij}	SUM
Sb1	O5	2.218	1.910	0.37	0.44	
	O5	2.218	1.910	0.37	0.44	
	O5	2.218	1.910	0.37	0.44	
	O5	2.218	1.910	0.37	0.44	
	O6	1.960	1.910	0.37	0.87	2.63
Ge1	O1	1.754	1.75	0.33	0.99	
	O1	1.754	1.75	0.33	0.99	
	O1	1.754	1.75	0.33	0.99	
	O1	1.754	1.75	0.33	0.99	3.96
V1	O2	1.965	1.803	0.852	0.65	
	O2	1.965	1.803	0.852	0.65	
	O2	1.965	1.803	0.852	0.65	
	O2	1.965	1.803	0.852	0.65	
	O7	1.560	1.803	0.852	1.93	4.53
Nb1	O1	2.439	1.911	0.852	0.24	
	O2	2.021	1.911	0.852	0.74	
	O2	2.021	1.911	0.852	0.74	
	O3	1.739	1.911	0.852	1.59	
	O4	1.925	1.911	0.852	0.96	
	O4	1.925	1.911	0.852	0.96	5.23
Nb2	O1	2.398	1.911	0.852	0.27	
	O4	1.957	1.911	0.852	0.88	
	O4	1.957	1.911	0.852	0.88	
	O4	1.957	1.911	0.852	0.88	
	O4	1.957	1.911	0.852	0.88	
	O5	1.787	1.911	0.852	1.40	5.19

Table S3. Bond valence summations of metal atoms of FZU-3H.

Atom1	Atom2	R_{ij}	R_o	B	S_{ij}	SUM
Sb1	O5	2.239	1.910	0.37	0.41	
	O5	2.239	1.910	0.37	0.41	
	O5	2.239	1.910	0.37	0.41	
	O5	2.239	1.910	0.37	0.41	
	O6	1.920	1.910	0.37	0.97	2.61
Ge1	O1	1.790	1.750	0.33	0.89	
	O1	1.790	1.750	0.33	0.89	
	O1	1.790	1.750	0.33	0.89	
	O1	1.790	1.750	0.33	0.89	3.56
Nb1	O2	1.957	1.911	0.852	0.88	
	O2	1.957	1.911	0.852	0.88	
	O3	1.757	1.911	0.852	1.52	
	O4	1.995	1.911	0.852	0.80	
	O4	1.995	1.911	0.852	0.80	
	O1	2.403	1.911	0.852	0.26	5.14
Nb2	O4	1.975	1.911	0.852	0.84	
	O4	1.975	1.911	0.852	0.84	
	O4	1.975	1.911	0.319	0.84	
	O4	1.975	1.911	0.852	0.84	
	O5	1.747	1.911	0.852	1.56	
	O1	2.380	1.911	0.852	0.28	5.20
V1	O2	2.027	1.803	0.852	0.55	
	O2	2.027	1.803	0.852	0.55	
	O2	2.027	1.803	0.852	0.55	
	O2	2.027	1.803	0.852	0.55	
	O7	1.640	1.803	0.852	1.55	3.75

Table S4. Bond valence summations of metal atoms of FZU-3A.

Atom1	Atom2	R_{ij}	R_o	*B*	S_{ij}	SUM
Sb1	O5	2.180	1.910	0.37	0.48	
	O5	2.180	1.910	0.37	0.48	
	O5	2.180	1.910	0.37	0.48	
	O5	2.180	1.910	0.37	0.48	
	O6	1.970	1.910	0.37	0.85	2.77
Ge1	O1	1.760	1.750	0.33	0.97	
	O1	1.760	1.750	0.33	0.97	
	O1	1.760	1.750	0.33	0.97	
	O1	1.760	1.750	0.33	0.97	3.88
Nb1	O2	1.994	1.911	0.852	0.80	
	O2	1.994	1.911	0.852	0.80	
	O3	1.746	1.911	0.852	1.56	
	O4	1.960	1.911	0.852	0.88	
	O4	1.960	1.911	0.852	0.88	
	O1	2.440	1.911	0.852	0.24	5.16
Nb2	O4	1.992	1.911	0.852	0.80	
	O4	1.992	1.911	0.852	0.80	
	O4	1.992	1.911	0.852	0.80	
	O4	1.992	1.911	0.852	0.80	
	O5	1.800	1.911	0.852	1.35	
	O1	2.380	1.911	0.852	0.27	4.82
V1	O2	1.949	1.803	0.852	0.67	
	O2	1.949	1.803	0.852	0.67	
	O2	1.949	1.803	0.852	0.67	
	O2	1.949	1.803	0.852	0.67	
	O7	1.600	1.803	0.852	1.73	4.41

Table S5. Bond valence summations of metal atoms of FZU-3B.

Atom1	Atom2	R_{ij}	R_o	B	S_{ij}	SUM
Sb1	O5	2.217	1.910	0.37	0.44	
	O5	2.217	1.910	0.37	0.44	
	O5	2.217	1.910	0.37	0.44	
	O5	2.217	1.910	0.37	0.44	
	O6	1.970	1.910	0.37	0.85	2.61
Ge1	O1	1.761	1.750	0.33	0.97	
	O1	1.761	1.750	0.33	0.97	
	O1	1.761	1.750	0.33	0.97	
	O1	1.761	1.750	0.33	0.97	3.88
Nb1	O2	1.980	1.911	0.852	0.83	
	O2	1.980	1.911	0.852	0.83	
	O3	1.729	1.911	0.852	1.64	
	O4	1.984	1.911	0.852	0.82	
	O4	1.984	1.911	0.852	0.82	
	O1	2.428	1.911	0.852	0.25	5.19
Nb2	O4	1.987	1.911	0.852	0.81	
	O4	1.987	1.911	0.852	0.81	
	O4	1.987	1.911	0.852	0.81	
	O4	1.987	1.911	0.852	0.81	
	O5	1.756	1.911	0.852	1.52	
	O1	2.380	1.911	0.852	0.28	5.04
V1	O2	1.990	1.803	0.852	0.60	
	O2	1.990	1.803	0.852	0.60	
	O2	1.990	1.803	0.852	0.60	
	O2	1.990	1.803	0.852	0.60	
	O7	1.590	1.803	0.852	1.78	4.18

Table S6. EA results of FZU-3 and FZU-3H.

Sample	N (%)*	C (%)*	H (%)
FZU-3	<0.30	<0.30	0.83
FZU-3H	<0.30	<0.30	0.83

*C and N contents below the instrument detection limits.

Table S7. Comparison of the electrochemical performances of Niobium-based electrodes for LIBs.

Materials	Specific capacity (mAh g^{-1})	Cycling performance	Ref.
**FZU-3H**	**519** (0.1 A g^{-1})	173 (1 A g^{-1} 1600 cycles)	This work
**FZU-3**	**387** (0.1 A g^{-1})	70 (1 A g^{-1} 930 cycles)	This work
N-C@-MSC-Nb_2O_5	225 (0.05 A g^{-1})	170 (2 A g^{-1} 1000 cycles)	[14]
NWO	221 (0.089 A g^{-1})	130 (1.78 A g^{-1} 4000 cycles)	[15]
$W_3Nb_{14}O_{44}$	121 (0.089 A g^{-1})	150 (3.6 A g^{-1} 1000 cycles)	[16]
S-Nb_2O_5@S-rGO	350 (0.01 A g^{-1})	130 (2 A g^{-1} 7000 cycles)	[17]
d-H-Nb_2O_5	225 (0.1 A g^{-1})	138 (2 A g^{-1} 2000 cycles)	[18]
TNO-OLC-CO_2	350 (0.04 A g^{-1})	240 (0.01 A g^{-1} 500 cycles)	[19]
Nb_2O_5-HGF-2.0	188 (0.2 A g^{-1})	100 (2 A g^{-1} 1000 cycles)	[20]
T-Nb_2O_5@C	205 (0.1 A g^{-1})	120 (2 A g^{-1} 2000 cycles)	[21]
Nb_2O_5@NC	203 (0.1 A g^{-1})	106 (2 A g^{-1} 2000 cycles)	[22]
Nb_2C-rGO-2.1	357 (0.05 A g^{-1})	260 (1 A g^{-1} 1000 cycles))	[23]

Table S8. Comparison of the electrochemical performances of POM-based electrodes for LIBs.

Materials	Specific capacity (mAh g^{-1})	Cycling performance	Ref.
**FZU-3H**	**519** (0.1 A g^{-1})	173 (1 A g^{-1} 1600 cycles)	This work
**FZU-3**	**387** (0.1 A g^{-1})	70 (1 A g^{-1} 930 cycles)	This work
$PMo_{10}V_2$	231 (1 C)	227 (1 C 50 cycles)	[24]
Ni_6PW_9	420 (0.1 A g^{-1})	350 (0.1 A g^{-1} 4000 cycles)	[25]
NENU-507	663 (0.1 A g^{-1})	662 (0.1 A g^{-1} 100 cycles)	[26]
PMo_{12}	225 (1 A g^{-1})	220 (1 A g^{-1} 200 cycles)	[27]
PANI- PMo_{12}	180 (0.1 C)	150 (0.1 C 50 cycles)	[28]
$Ni_{2.5}PW_9$	325 (0.1 C)	141 (1 C 50 cycles)	[29]
BAS-11	200 (0.1 A g^{-1})	150 (0.1 A g^{-1} 300 cycles)	[30]
γ-LiV_2O_5-ESD	250 (0.5 C)	234 (0.5 C 100 cycles)	[31]

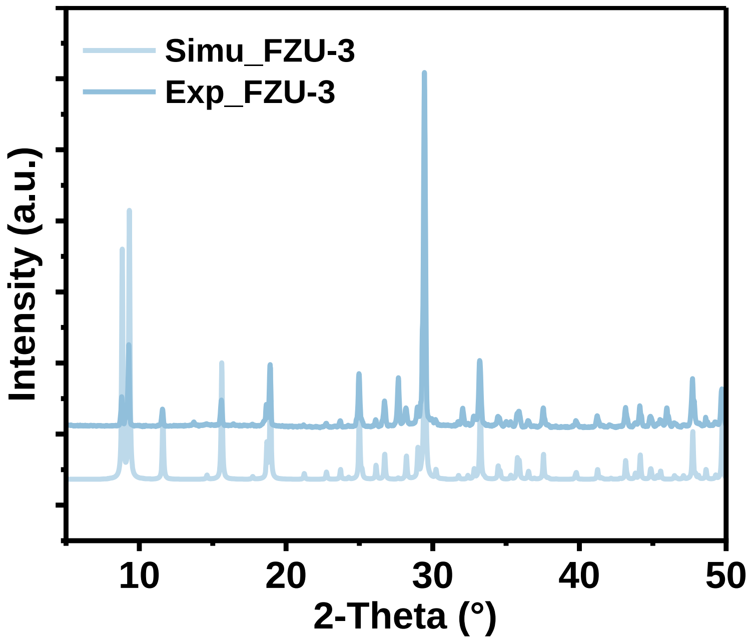

Figure S1. Simulated and experimental PXRD patterns of FZU-3.

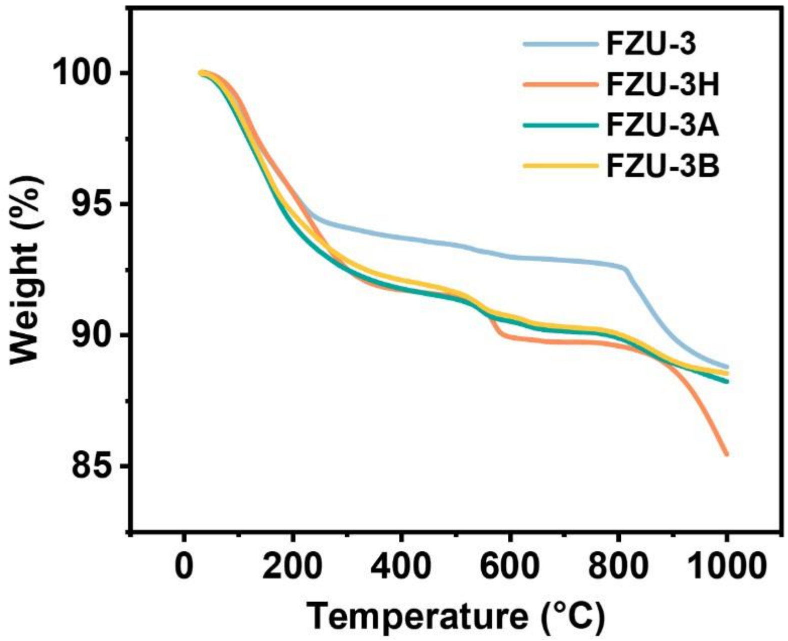

Figure S2. TG curves of FZU-3, FZU-3H, FZU-3A and FZU-3B.

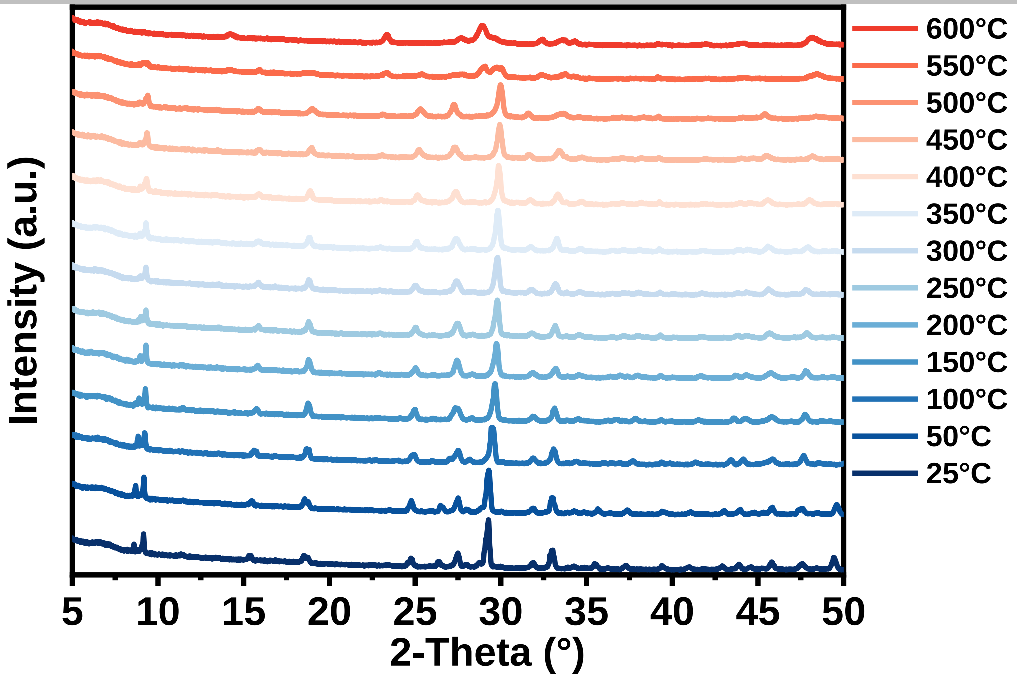

Figure S3. In-situ variable temperature PXRD patterns of FZU-3.

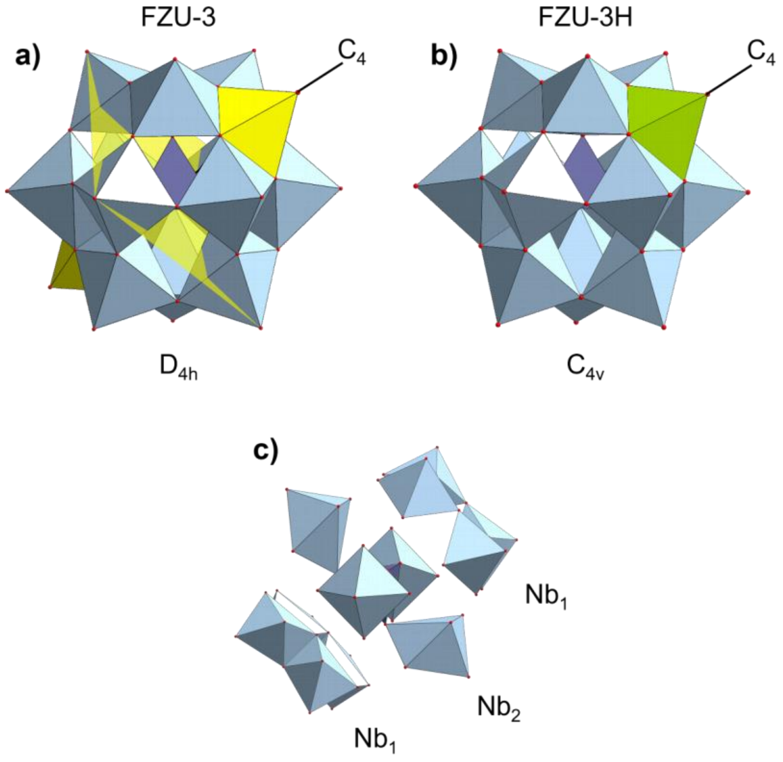

Figure S4. SUBs picture of FZU-3 (a) and FZU-3H (b). Polyhedra key: NbO_6, azure; VO_5, yellow and grass green; GeO_4, deep purple.

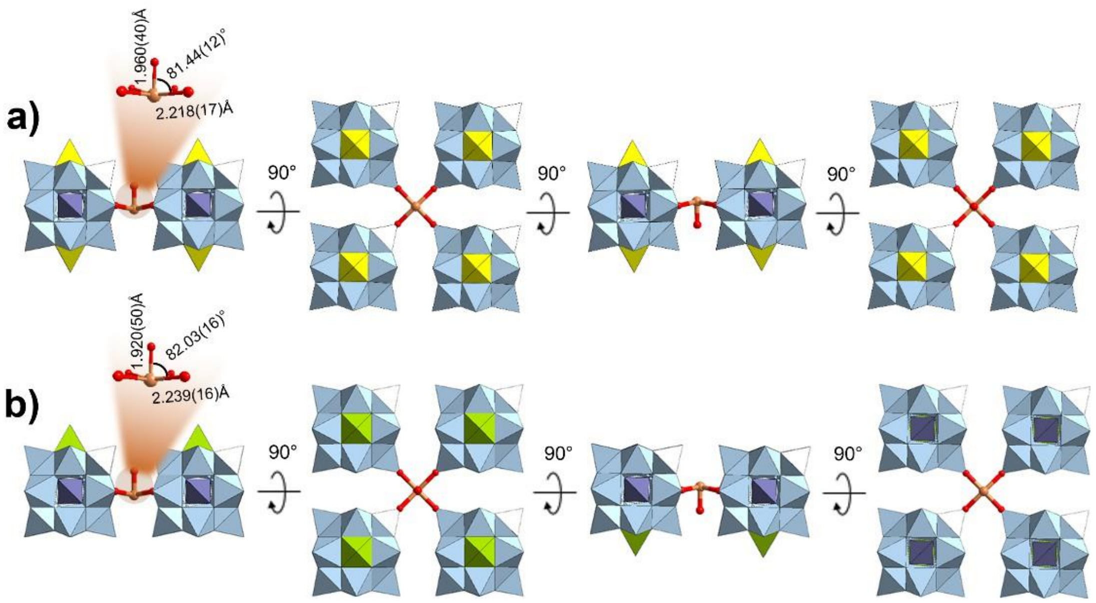

Figure S5. Coordination modes of Sb atom of FZU-3 (a) and FZU-3H (b). The coordination modes of the Sb atom at different angles. Polyhedra key: NbO_6, azure; VO_5, yellow and grass green; GeO_4, deep purple.

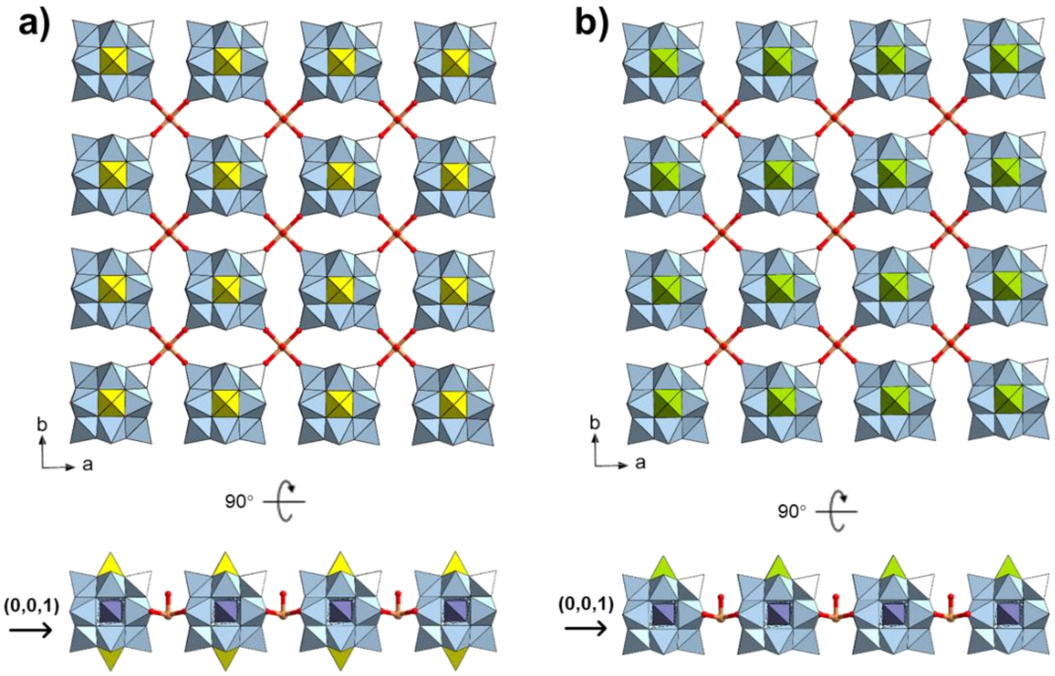

Figure S6. The ab plane of FZU-3 (a) and FZU-3H (b). Polyhedra key: NbO_6, azure; VO_5, yellow and grass green; GeO_4, deep purple.

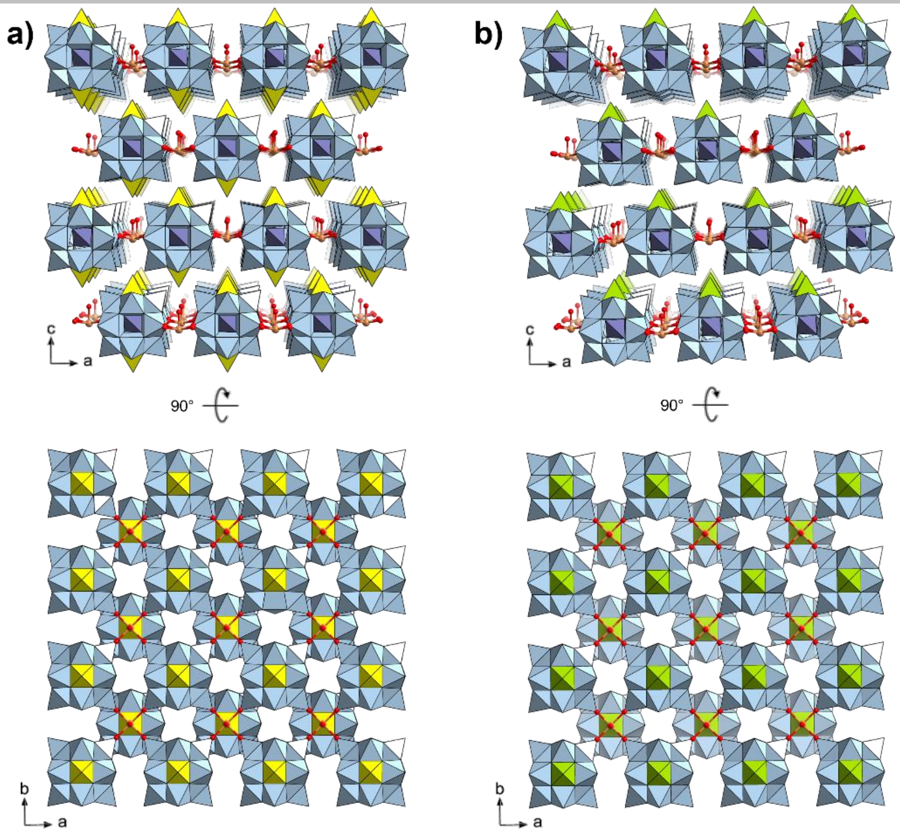

Figure S7. The three-dimensional structure of FZU-3 (a) and FZU-3H (b). Polyhedra key: NbO_6, azure; VO_5, yellow and grass green; VO_4, yellow; GeO_4, deep purple.

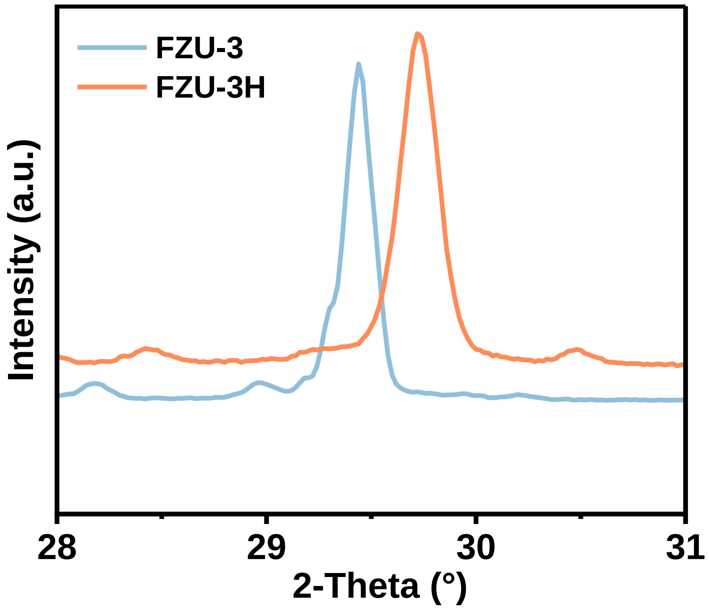

Figure S8. PXRD spectra of FZU-3 and FZU-3H.

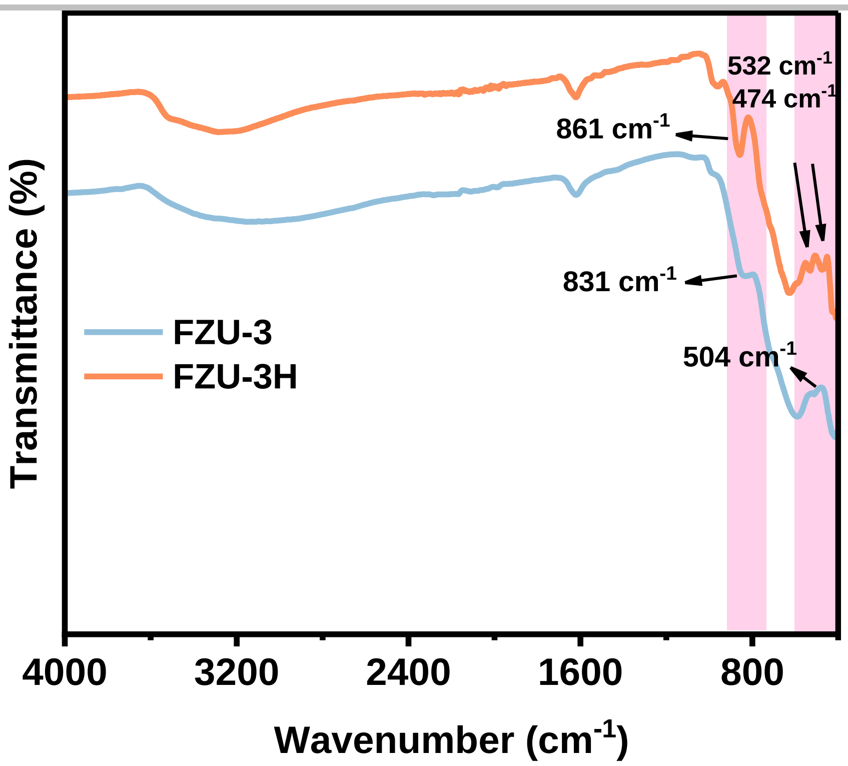

Figure S9. FT-IR spectra of FZU-3 and FZU-3H.Note: In the IR spectrum, the broad peak at 3243 cm^{-1} is attributed to the v(O-H) stretching vibration of water. The v(C-H) bending vibrations peak appear at about 1600 cm^{-1}. The characteristic peaks in the range of 980-960 cm^{-1} and 526-507 cm^{-1} can be attributed to the stretching vibrations of v(V=O) and stretching vibrations of v(V-O). The peaks that appear in the range of 400-1000 cm^{-1} can be attributed to the characteristic absorption peak of Nb-O, of which 880-800 cm^{-1} is the stretching vibration peak of v(Nb-O_t) and 676 cm^{-1} and 474 cm^{-1} are the stretching vibration peaks of v(Nb-O_b-Nb).

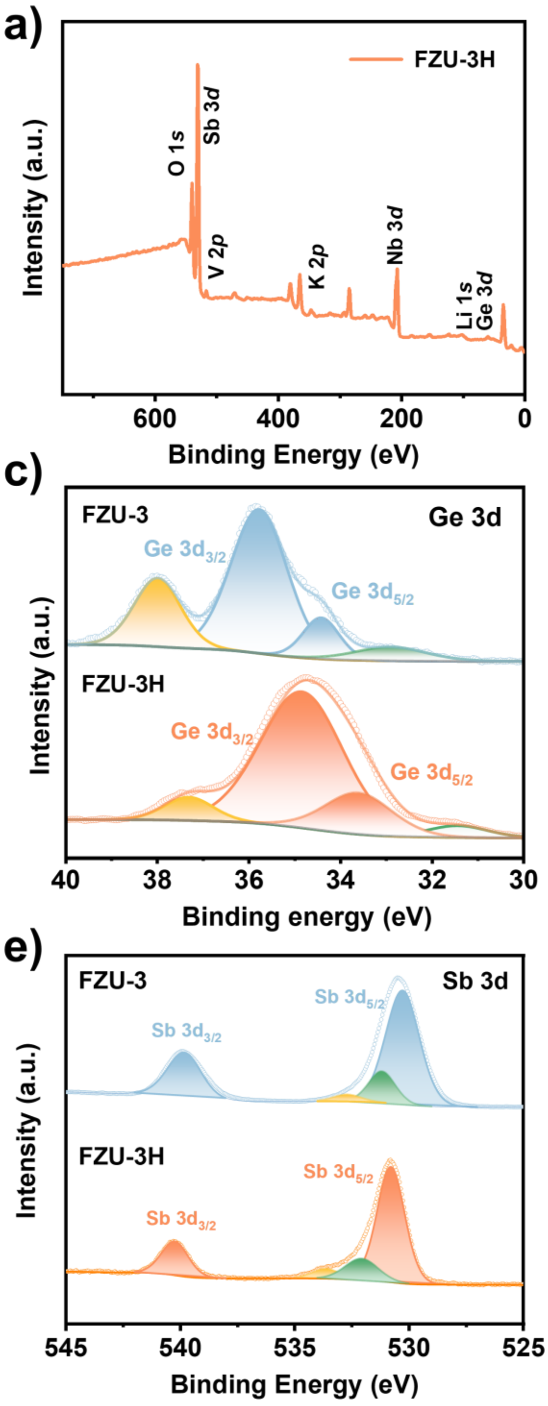

Figure S10. XPS survey spectra of FZU-3 and FZU-3H.

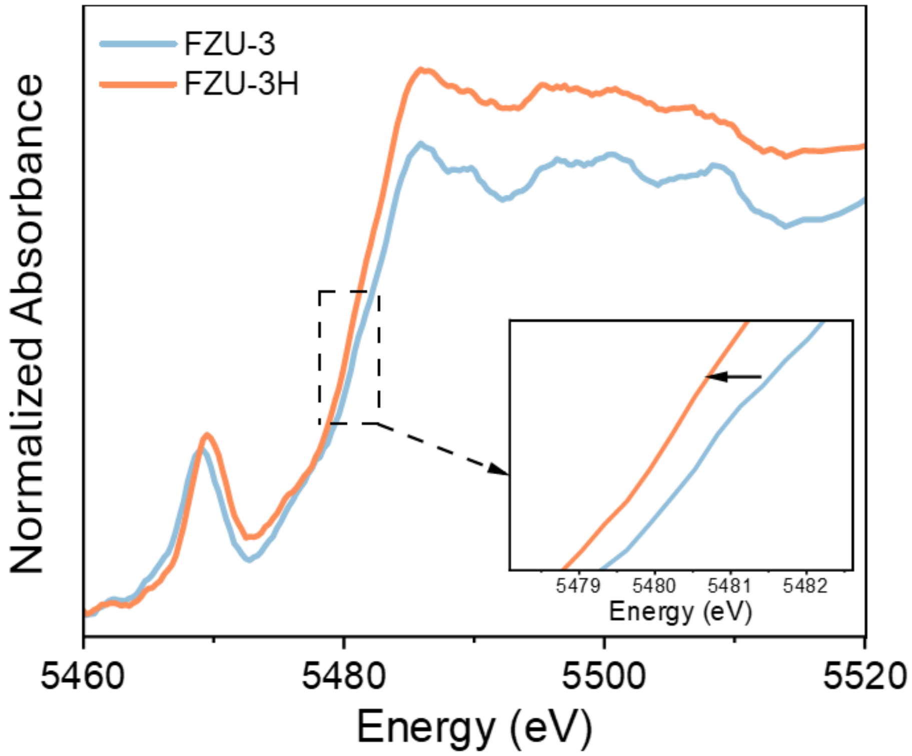

Figure S11. XAS of V K-edge in FZU-3 and FZU-3H.

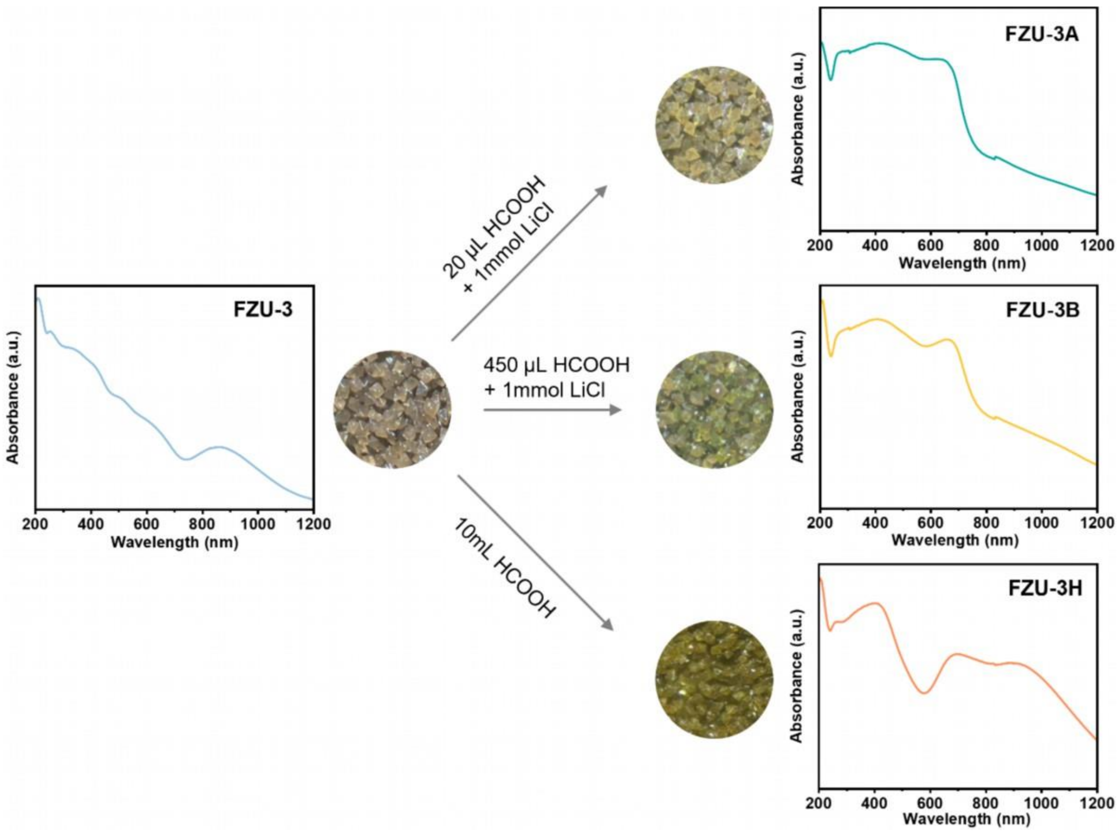

Figure S12. UV absorption spectra of FZU-3, FZU-3A, FZU-3B and FZU-3H and their corresponding optical images.

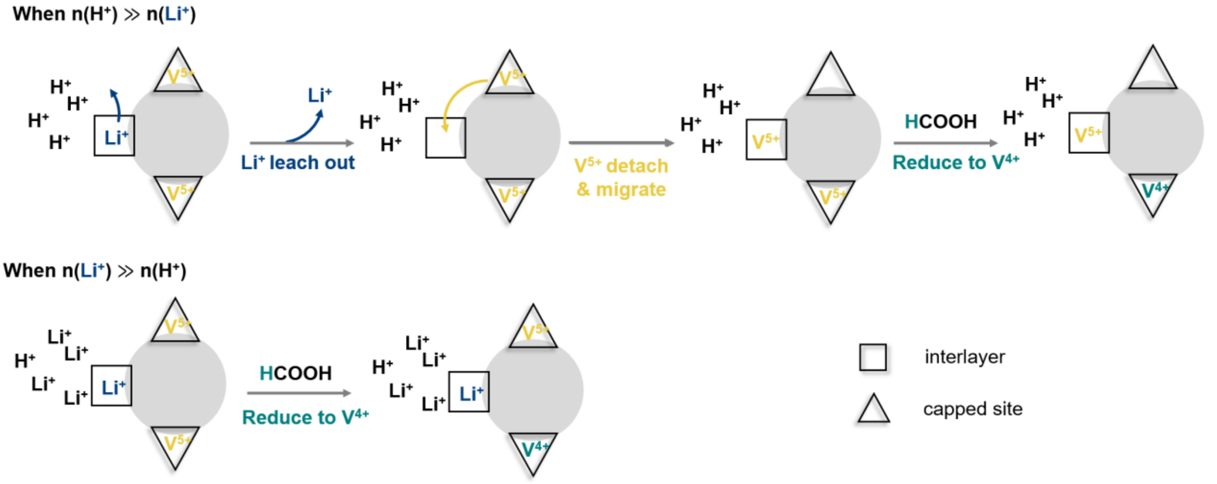

Figure S13. A possible HCOOH-induced SCSC transformation mechanism.

We propose that the detachment of capped vanadium is not due to reduction, based on the following data. Bond valence sum (BVS) calculations indicate that the oxidation state of the 0.5 capped V is +4. Moreover, literatures support the frequent occurrence of a mixed valence state (+4/+5) for capped V [32]. The tetrahedral V often exists in its highest oxidation state Therefore, the reduction of capped V cannot be considered the driving force for its detachment.

We propose a possible formic acid-induced single-crystal-to-single-crystal (SCSC) transformation mechanism (Figure S13, also show above). First, formic acid, being acidic, dissociates to release a large number of protons. These protons leach out monovalent Li^+ ions from FZU-3. This process leads to the detachment of the capped V. Meanwhile, the V^{5+} at the cap site that has not left is reduced to V^{4+} by formic acid, and the reduced V^{4+} ion remains at the cap site.

Additionally, the experiment on formic acid-induced SCSC transformation in the presence of Li^+ further supports the reliability of this proposed mechanism. Firstly, we immersed the FZU-3 crystals in 10 mL of a 0.1 mol/L LiCl solution (1 mmol LiCl) and added 20 µL (0.029 mmol) formic acid. After soaking for 15 minutes, we obtained the crystal FZU-3A. SXRD analysis revealed that the presence of Li^+ in the solution prevented the leaching of monovalent Li^+ from FZU-3A structure. Consequently, the capped vanadium atoms did not detach. Meanwhile, a small amount of capped vanadium ions were reduced to the +4 oxidation state. Although the bond valence calculation results for the capped vanadium remained nearly unchanged, the reduction process was confirmed by the green coloration of the sample and the UV-visible absorption spectral data (Figure S12). This further confirms that the reduction of capped vanadium alone does not lead to its detachment.

Subsequently, while keeping the LiCl content constant, the amount of formic acid was increased to 450 µL, resulting in the formation of crystal FZU-3B. Its structure is similar to that of FZU-3H; however, compared to FZU-3H, FZU-3B exhibits lower leached lithium content, lower amount of vanadium detached from the cap, and a lower proportion of reduced capped vanadium (Figure S13). These findings indicate that increasing the amount of formic acid promotes the leaching of lithium and the detachment of high-valence vanadium.

In summary, a high concentration of formic acid enables the leaching of Li^+ ions and induces the migration of high-valence V^{5+}, while also reducing the capped V. Conversely, when the formic acid content is extremely low and abundant Li^+ ions are present, the low H^+ concentration is insufficient to leach Li^+ ions. In this case, the capped V remains attached, although it is still reduced.

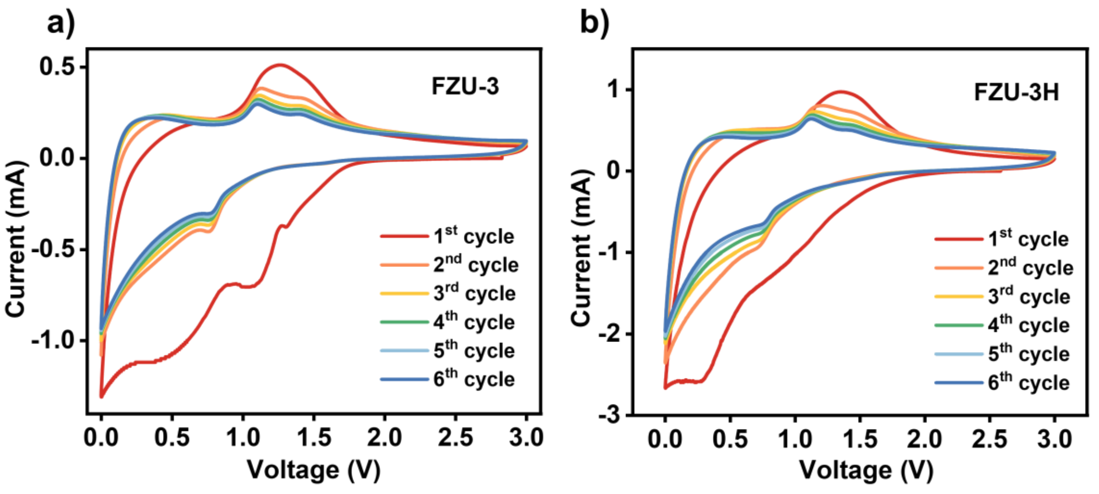

Figure S14. CV curves of FZU-3 (a) and FZU-3H (b) at a scan rate of 1.0 mV s^{-1}.

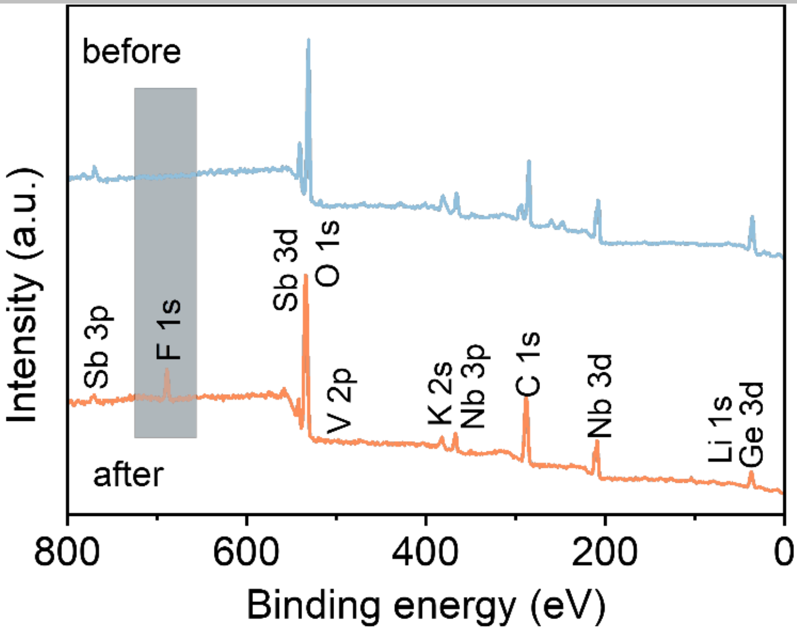

Figure S15. XPS spectra of FZU-3H before and after the first cycle.

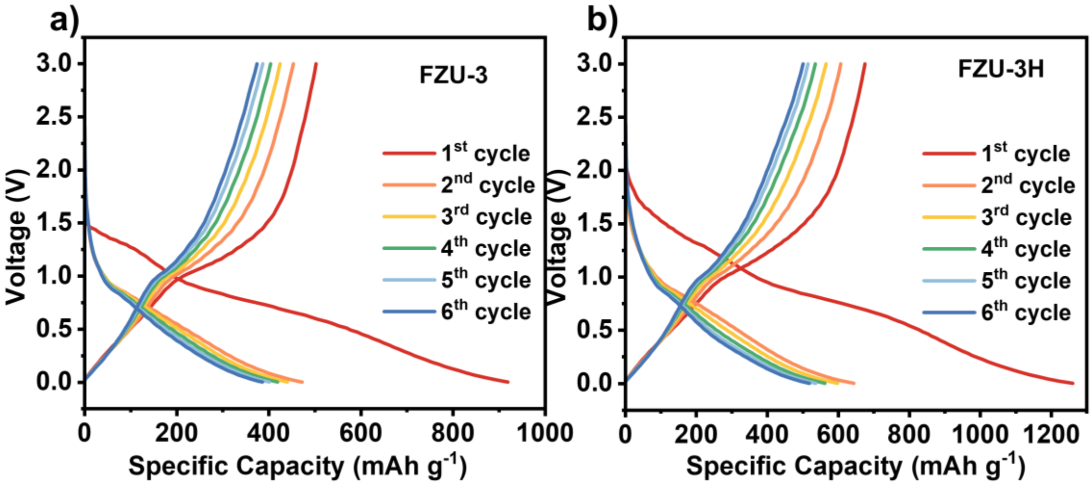

Figure S16. Galvanostatic charging-discharging curves of FZU-3 (a) and FZU-3H (b) at 0.1 A g^{-1}.

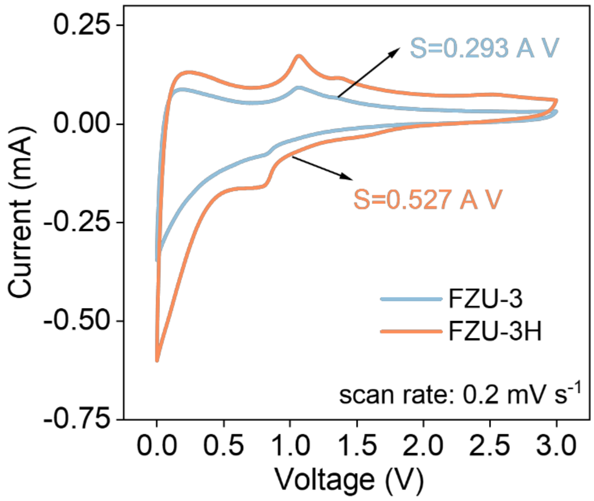

Figure S17. CV comparison of FZU-3 electrode and FZU-3H electrode at a scan rate of 0.2 mV s^{-1}.

Note: We calculated the number of Li^+ ions that can be incorporated into the two crystals from the CV curves at 0.2 mV s^{-1}. The FZU-3H electrode reaches a specific capacity of 504.4 mAh g^{-1} (accommodated 41 Li^+ ions), while the FZU-3 only has a 377.4 mAh g^{-1} (accommodated 32 Li^+ ions).

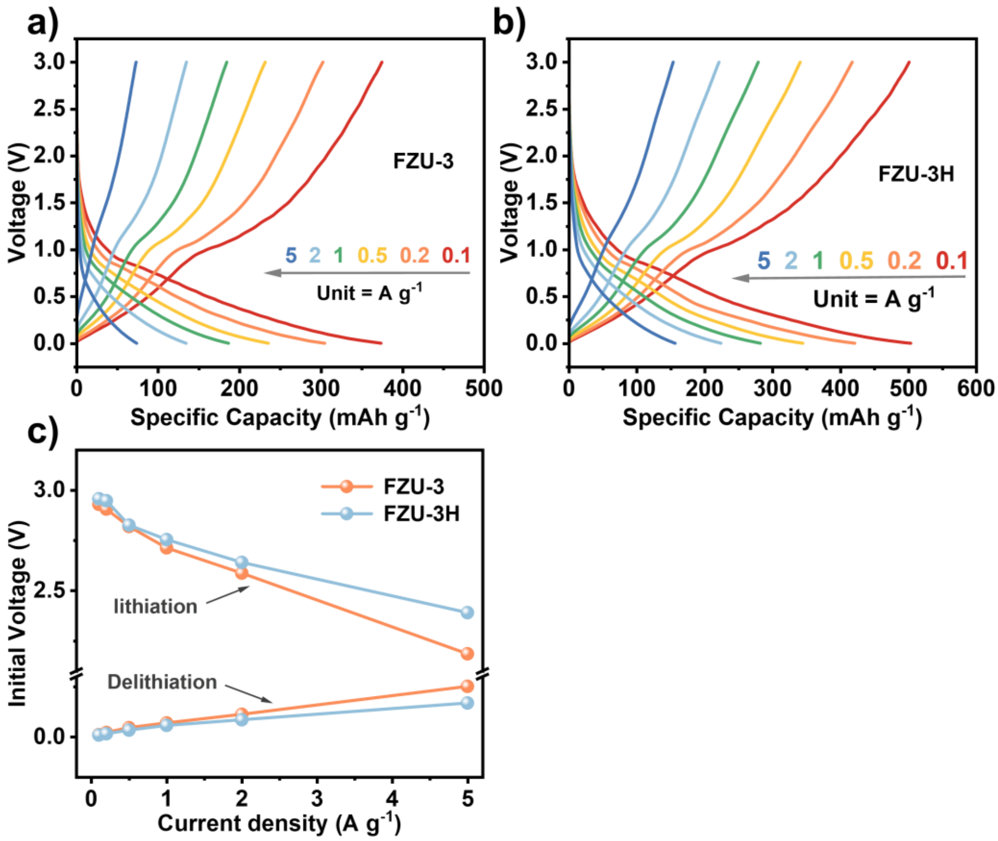

Figure S18. Charge-discharge curves of FZU-3 (a) and FZU-3H (b) at different current densities. (c) The initial voltages of lithiation/delithiation at different current densities for FZU-3 and FZU-3H electrodes.

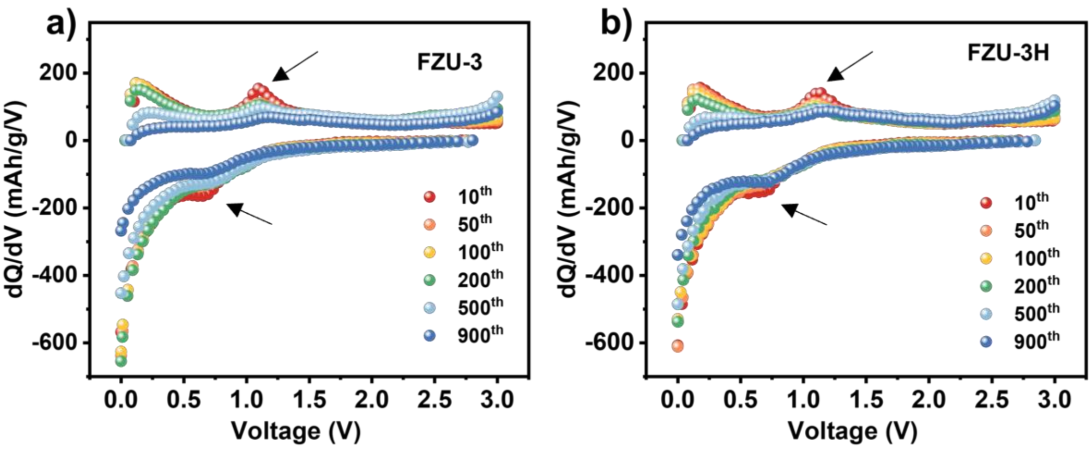

Figure S19. Differential charge versus voltage curves of FZU-3 (a) and FZU-3H (b) Note: As the number of cycling laps increases, the peak intensity of FZU-3 decreases more significantly compared to FZU-3H, indicating a greater percentage loss of active material in the electrode, and the peak position is also shifted more significantly, suggesting that insertion of Li^+ in FZU-3 is more difficult.

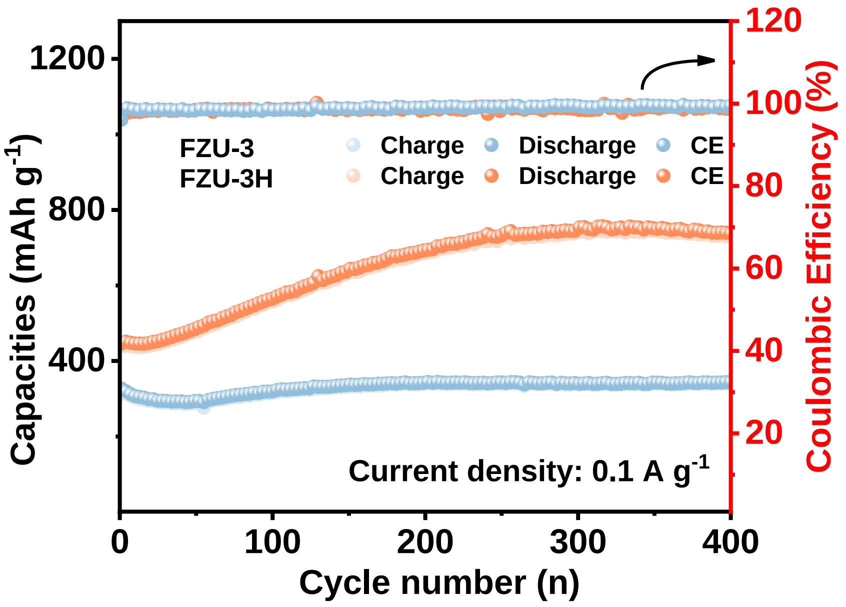

Figure S20. Cycling stability of FZU-3 and FZU-3H at 0.1 A g^{-1}.Note: With the wettability enhanced, the charge/discharge capacity of FZU-3H shows a slow upward trend, the capacity retention increases to 154% at 400th.

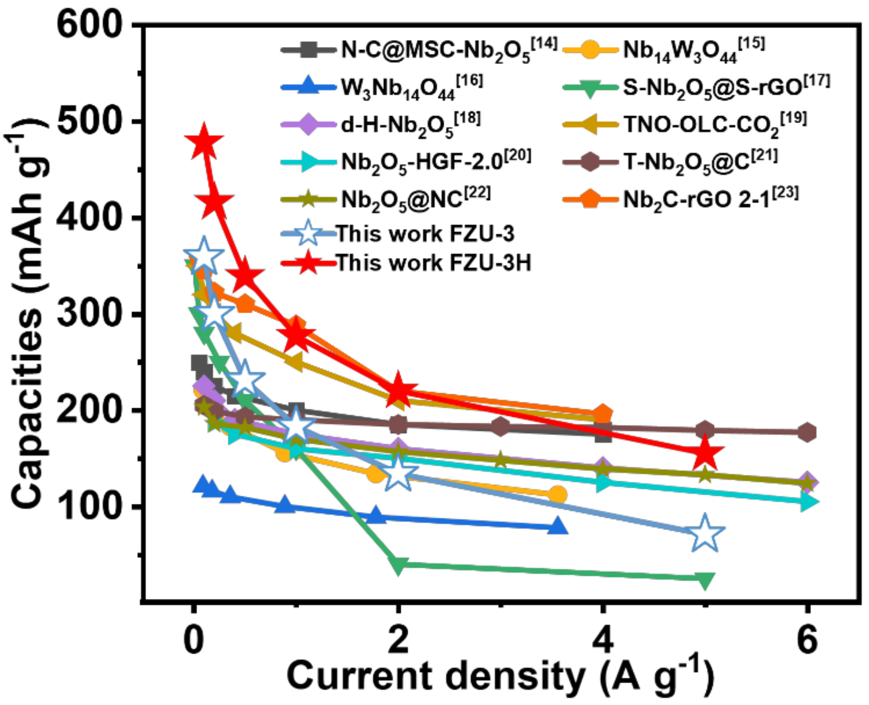

Figure S21. Performance comparison of FZU-3H and FZU-3 with other reported materials.

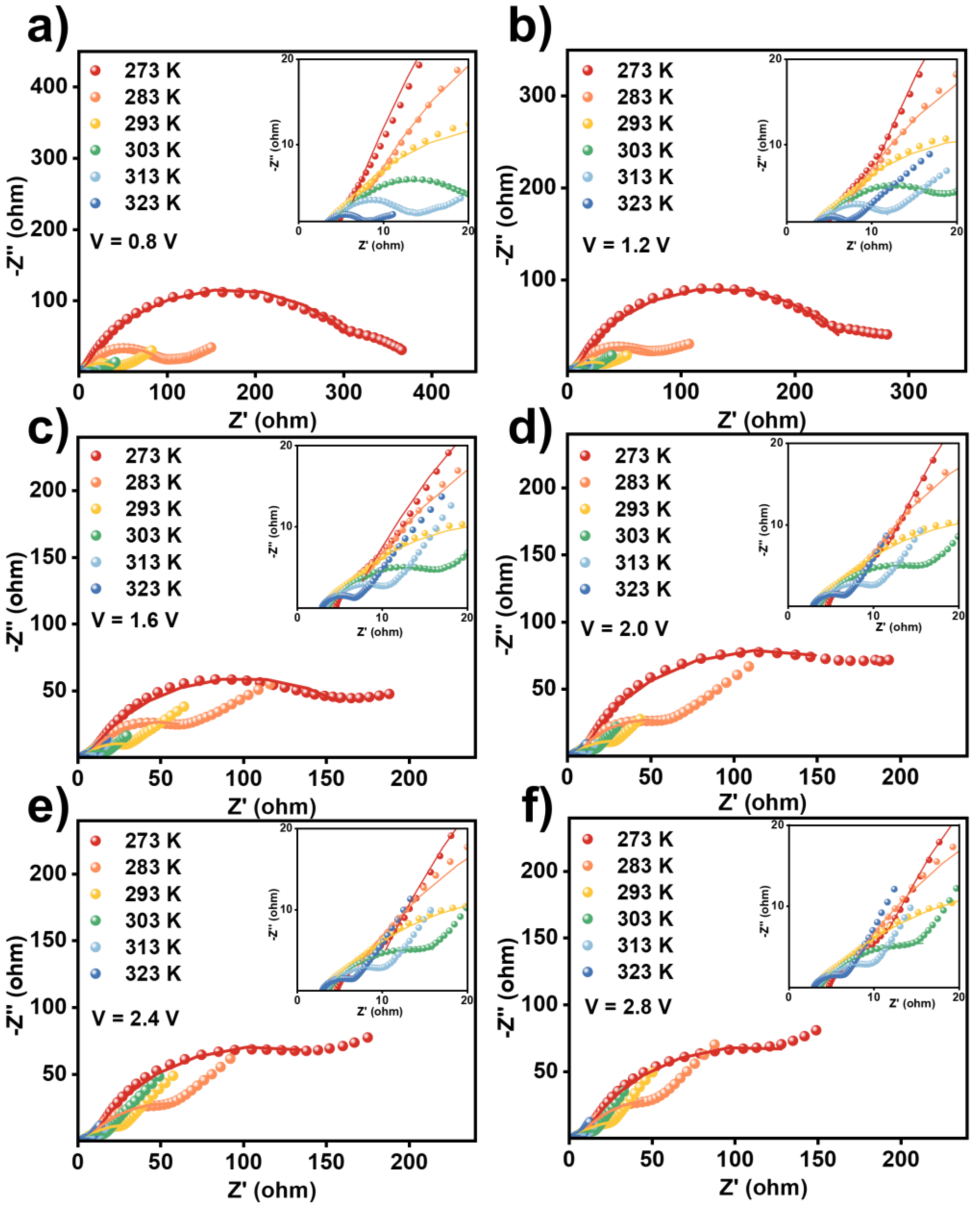

Figure S22. The EIS spectra of the FZU-3 electrode under different temperatures from 0.8 V to 2.8 V. The insets are the local magnification profiles.

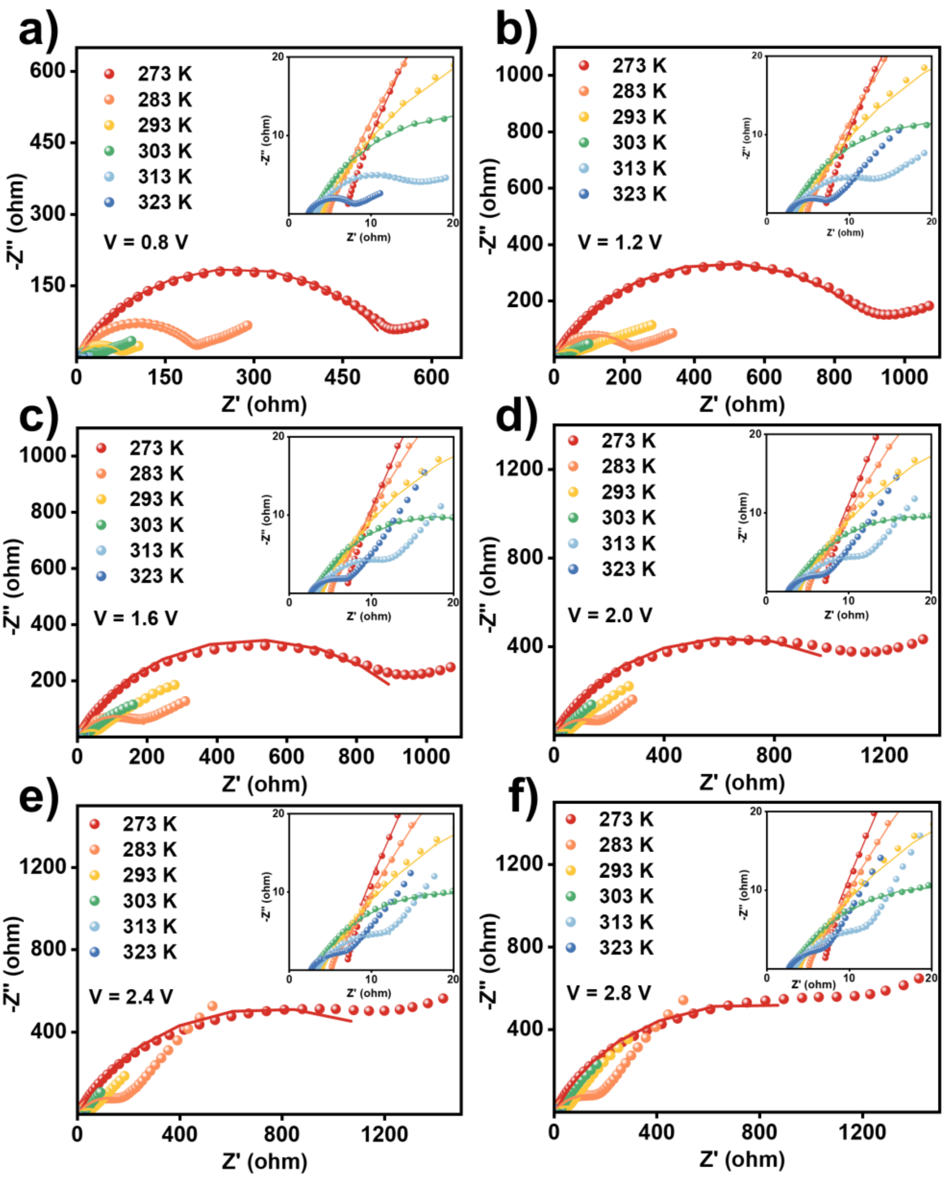

Figure S23. The EIS spectra of the FZU-3H electrode under different temperatures from 0.8 V to 2.8 V. The insets are the local magnification profiles.

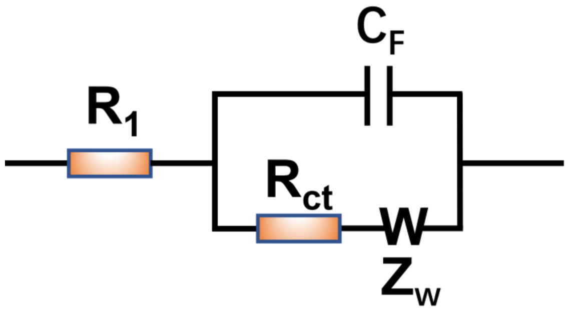

Figure S24. The equivalent circuit is used in fitting the electrochemical impedance spectroscopy (EIS).

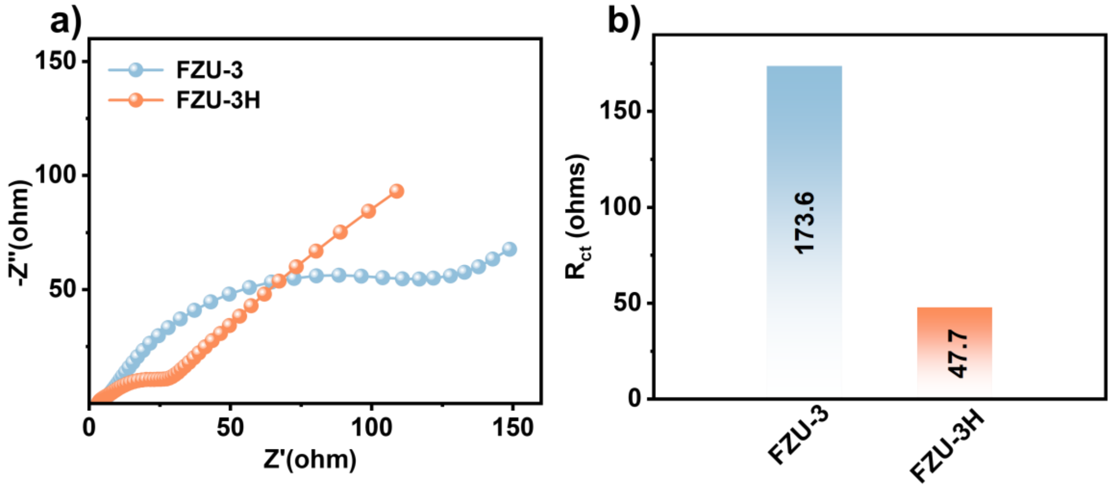

Figure S25. (a) EIS of the FZU-3 and FZU-3H electrodes before cycling. (b) Charge transfer resistance (R_{ct}) of FZU-3 and FZU-3H electrodes.

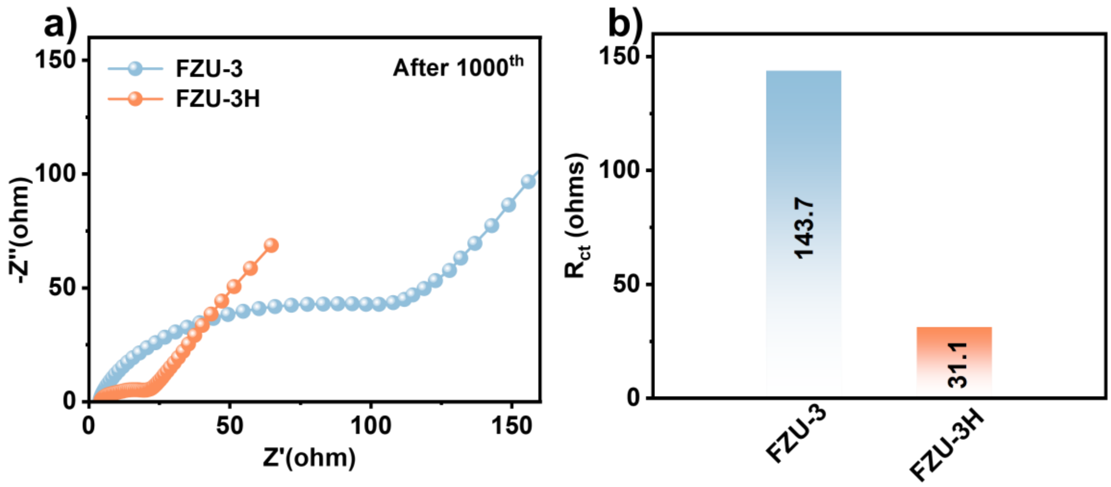

Figure S26. (a) EIS of the FZU-3 and FZU-3H electrodes after 1000 charge-discharge cycles at 1.0 A g^{-1}. (b) Charge transfer resistance (R_{ct}) of FZU-3 and FZU-3H electrodes.

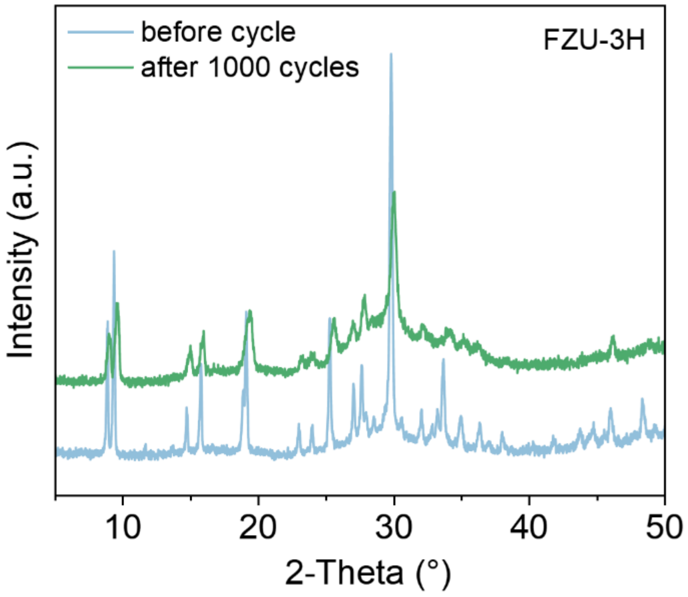

Figure S27. XRD of the FZU-3H electrodes after 1000 charge-discharge cycles at 1.0 A g^{-1}.

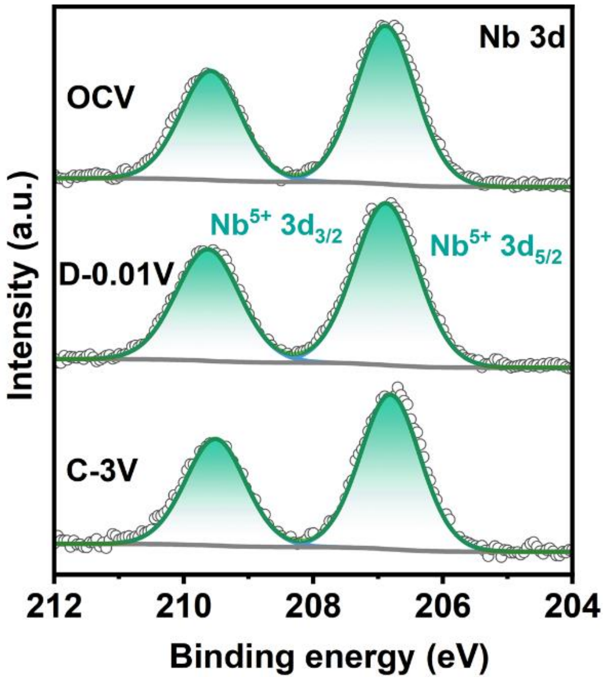

Figure S28. Nb3d XPS spectra of FZU-3H electrode at different charge/discharge stages.

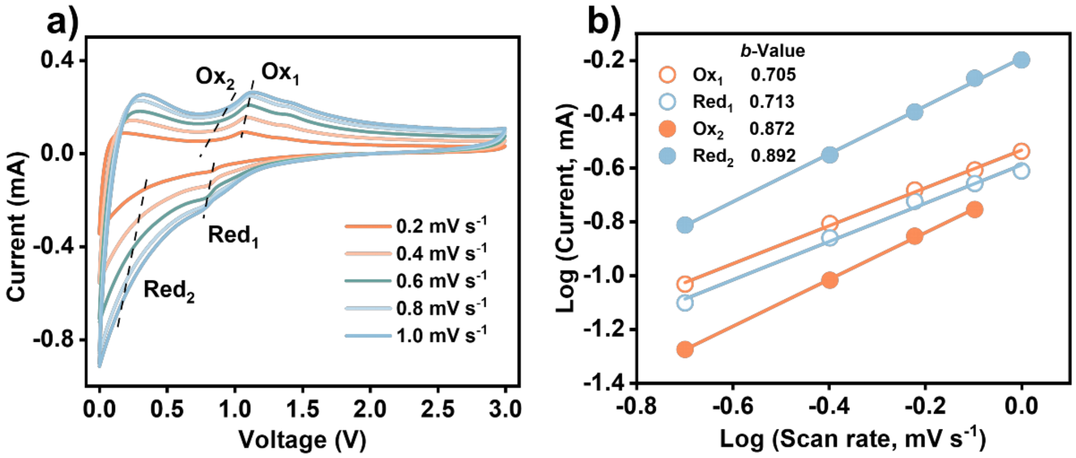

Figure S29. CV curves of FZU-3 at different scan rates. (b) Plot the peak currents and logarithmic scan rates of FZU-3 at different scan rates.

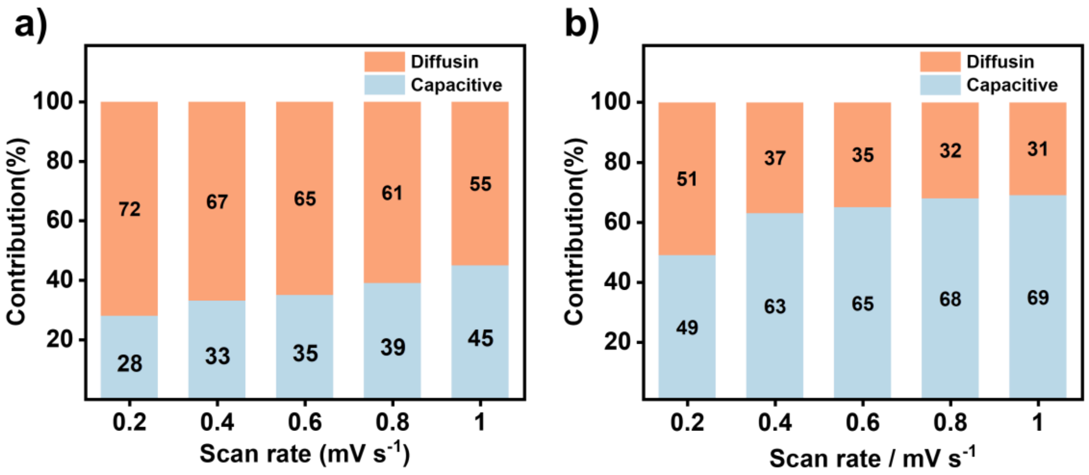

Figure S30. Proportion of diffusion and capacitance of FZU-3 (a) and FZU-3H (b) at different scan rates.

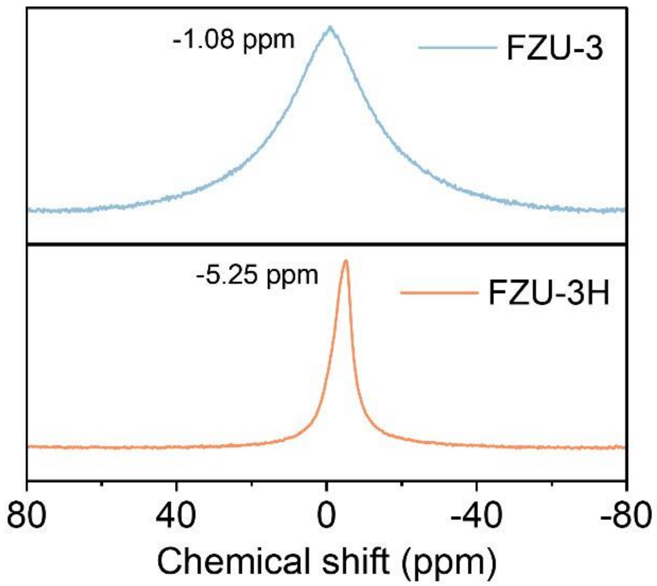

Figure S31. Solid state ^{7}Li NMR of FZU-3 and FZU-3H.

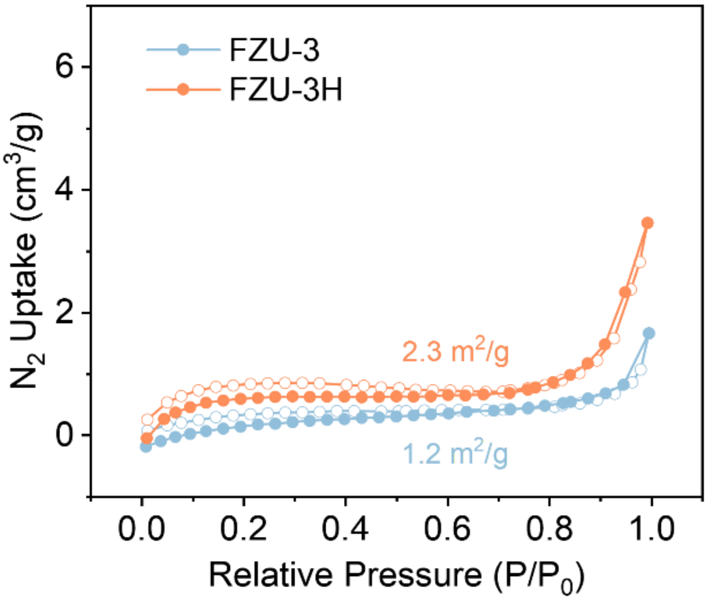

Figure S32. N_2 adsorption/desorption curves of FZU-3 and FZU-3H.

References

[1] M. Filowitz, RKC Ho, W.G. Klemperer, W. Shum, Inorg. Chem. **1979**, 18, 93-103.

[2] a) H. Mistry, A. S. Varela, C. S. Bonifacio, I. Zegkinoglou, I. Sinev, Y.-W. Choi, K. Kisslinger, E. A. Stach, J. C. Yang, P. Strasser, *Nat. Commun.* **2016**, *7*, 12123; b) J.-Y. Kim, D. Hong, J.-C. Lee, H. G. Kim, S. Lee, S. Shin, B. Kim, H. Lee, M. Kim, J. Oh, *Nat. Commun.* **2021**, *12*, 3765.

[3] X. Hu, M. Qiu, Y. Liu, J. Yuan, J. Chen, H. Zhan, Z. Wen, *Adva. Energy Mater.* **2022**, *12*, 2202318- 2202331.

[4] I. D. Brown, D. Altermatt. *Acta Cryst*. (**1985**). B41, 244-247.

[5] Evans, H. T. Z. Kristallogr., Kristallgeom. Kristallphys., Kristallchem. **1960** 14, 257-277.

[6] a) D. H. Guan, X. X. Wang, L. N. Song, C. L. Miao, J. Y. Li, X. Y. Yuan, X. Y. Ma, J. J. Xu, *Angew. Chem. Int. Ed.* **2024***, 63, e202317949*; b) S. Zhang, F. Zhao, J. Chen, J. Fu, J. Luo, S. H. Alahakoon, L.-Y. Chang, R. Feng, M. Shakouri, J. Liang, Y. Zhao, X. Li, L. He, Y. Huang, T.-K. Sham, X. Sun, Nat. Commun. **2023**, 14, 3780.

[7] a) M. Amiri, N. P. Martin, C. L. Feng, J. K. Lovio, M. Nyman, *Angew. Chem. Int. Ed.* **2021**, 60, 12461-12466; b) K. Yonesato, H. Ito, H. Itakura, D. Yokogawa, T. Kikuchi, N. Mizuno, K. Yamaguchi, K. Suzuki, *J. Am. Chem. Soc.* **2019**, *141*, 19550-19554; c) Q. Zheng, M. Kupper, W. Xuan, H. Oki, R. Tsunashima, D.-L. Long, L. Cronin, J. Am. Chem. Soc. **2019**, 141, 13479-13486; d) Q. Zheng, L. Vilà-Nadal, Z. Lang, J.-J. Chen, D.-L. Long, J. S. Mathieson, J. M. Poblet, L. Cronin, J. Am. Chem. Soc. **2018**, 140, 2595-2601.

[8] Tytko, K.H., Mehmke, J., Kurad, D. (**1999**). Bond length-bond valence relationships with particular reference to polyoxometalate chemistry. In: Bard, A.J., et al. Bonding and Charge Distribution in Polyoxometalates: A Bond Valence Approach. Structure and Bonding, vol 93. Springer, Berlin, Heidelberg.

[9] X. C. Ren, J. S. Wang, D. M. Zhu, Q. W. Li, W. F. Tian, L. Wang, J. B. Zhang, L. Miao, P. K. Chu, K. F. Huo, *Nano Energy* **2018**, *54*, 322-330.

[10] W. Wang, P. Li, H. Zheng, Q. Liu, F. Lv, J. Wu, H. Wang, S. Guo, *Small* **2017**, *13*, 1702228-1702235.

[11] J. P. Perdew, K. Burke, M. Ernzerhof, *Phys. Rev. Lett.* **1996**, *77*, 3865-3868.

[12] a) P. E. Blöchl, *Phys. Rev. B* **1994**, *50*, 17953-17979; b) G. Kresse, D. Joubert, *Phys. Rev. B* **1999**, *59*, 1758-1775.

[13] G. Henkelman, B. P. Uberuaga, H. Jónsson, *J. Chem. Phys.* **2000**, *113*, 9901-9904.

[14] Z. Song, H. Li, W. Liu, H. Zhang, J. Yan, Y. Tang, J. Huang, H. Zhang, X. Li, *Adv. Mater.* **2020**, *32*, 2001001-2001010.

[15] Y. Yang, H. Zhu, J. Xiao, H. Geng, Y. Zhang, J. Zhao, G. Li, X. L. Wang, C. C. Li, Q. Liu, *Adv. Mater.* **2020**, *32*, 2408923- 2408938.

[16] W. T. Yao, H. J. Zhu, M. Wang, P. H. Li, P. Liu, P. C. Zou, A. M. Nie, G. Z. Wang, F. Y. Kang, C. Yang, *Chem. Mater.* **2022**, *34*, 388-398.

[17] Q. Deng, Y. Fu, C. Zhu, Y. Yu, *Small* **2019**, *15*, 1804884-1804910.

[18] T. Li, G. Nam, K. Liu, J.-H. Wang, B. Zhao, Y. Ding, L. Soule, M. Avdeev, Z. Luo, W. Zhang, T. Yuan, P. Jing, M. G. Kim, Y. Song, M. Liu, *Energy Environ. Sci.* **2022**, *15*, 254-264.

[19] Ö. Budak, P. Srimuk, M. Aslan, H. Shim, L. Borchardt, V. Presser, *ChemSusChem* **2020**, *14*, 398-407.

[20] H. Sun, L. Mei, J. Liang, Z. Zhao, C. Lee, H. Fei, M. Ding, J. Lau, M. Li, C. Wang, X. Xu, G. Hao, B. Papandrea, I. Shakir, B. Dunn, Y. Huang, X. Duan, *Science* **2017**, *356*, 599-604.

[21] J. Meng, Q. He, L. Xu, X. Zhang, F. Liu, X. Wang, Q. Li, X. Xu, G. Zhang, C. Niu, Z. Xiao, Z. Liu, Z. Zhu, Y. Zhao, L. Mai, *Adv. Energy Mater.* **2019**, *9*, 1802695-1802706.

[22] F. Liu, Z. Zhu, Y. Chen, J. Meng, H. Wang, R. Yu, X. Hong, J. Wu, *ACS Appl. Mater. Interfaces* **2022**, *14*, 49865-49874.

[23] R. Butt, A. H. Siddique, S. W. Bokhari, S. Jiang, D. Lei, X. Zhou, Z. Liu, *Int. J. Energy Res.* **2019**, *43*, 4995-5003.

[24] M. Priyadarshini, S. Shanmugan, K. P. Kirubakaran, A. Thomas, M. Prakash, C. Senthil, C. W. Lee, K. Vediappan, *J. Phys. Chem. Solids* **2020**, *142,* 109468.

[25] Y. Yue, Y. Li, Z. Bi, G. M. Veith, C. A. Bridges, B. Guo, J. Chen, D. R. Mullins, S. P. Surwade, S. M. Mahurin, H. Liu, M. P. Paranthaman, S. Dai, *J. of Mater. Chem. A* **2015**, *3*, 22989-22995.

[26] Y.-Y. Wang, M. Zhang, S.-L. Li, S.-R. Zhang, W. Xie, J.-S. Qin, Z.-M. Su, Y.-Q. Lan, *Chem. Commun.* **2017**, *53*, 5204-5207.

[27] X. Jia, J. Wang, H. Hu, Y. F. Song, *Chem. – A Eur. J.* **2020**, *26*, 5257-5263.

[28] H. X. Yang, T. Song, L. Liu, A. Devadoss, F. Xia, H. Han, H. Park, W. Sigmund, K. Kwon, U. Paik, *J. Phys. Chem. C* **2013**, *117*, 17376-17381.

[29] T. Eren, N. Atar, M. L. Yola, H. Karimi-Maleh, A. T. Çolak, A. Olgun, *Ionics* **2015**, *21*, 2193-2199.

[30] S. Sun, L. Cui, K. Yu, M. Wang, J. Lv, S. Ge, B. Zhou, *ACS Appl. Nano Mater.* **2023**, *7*, 1310-1318.

[31] A. Bayaguud, Z. Zhang, M. Geng, Y. Fu, Y. Yu, C. Zhu, *Small Methods* **2019**, *3* 1900187.

[32] a) N. I. Gumerova, A. Rompel, *Nat. Rev. Chem.* **2018**, *2*, 0112; b) Q. Lan, Z.-M. Zhang, Y.-G. Li, Y. Lu, E.-B. Wang, *Dalton Trans.* **2014**, *43*, 16265-16269; c) G. Guo, Y. Xu, J. Cao, C. Hu, *Chem. Commu.* **2011**, *47*, 9411-9413; d) A. S. Assran, N. V. Izarova, U. Kortz, *CrystEngComm* **2010**, *12*, 2684-2686.
